# Supplementary material for: Pyramiding of bacterial blight resistance genes into promising restorer BRRI31R line through marker-assisted backcross breeding and evaluation of agro-morphological and physiochemical characteristics of developed resistant restorer lines
Source: PLoS One. 2024 Jun 12;19(6):e0301342. doi: 10.1371/journal.pone.0301342 (PMC11168670; doi:10.1371/journal.pone.0301342)
Supplement: S1 Table — (DOCX) [file pone.0301342.s001.docx]

| **S1 Table. PCR profile for gene-linked/ specific primers against *Xa4, xa5 xa13* and *Xa21* genes.** | | | | | | |
| --- | --- | --- | --- | --- | --- | --- |
| **Step** | **Cycling conditions** | **Temperature** | ***Xa4*** | ***xa5*** | ***xa13*** | ***Xa21*** |
| **I** | Initial Denaturation | 94^0^C | 4 min | 4 min | 4 min | 4 min |
| **II** | Denaturation | 94^0^C | 1 min | 1 min | 1 min | 30 sec |
| **III** | Annealing | 55^0^C | 1 min | 1 min | 1 min | 30 sec |
| **IV** | Extension | 72^0^C | 2 min | 1 min | 1 min | 1 min |
| **V** | Go to Step II |  | 35 cycles | 35 cycles | 35 cycles | 30 cycles |
| **VI** | Final Extension | 72^0^C | 8 min | 7 min | 7 min | 8 min |
| **VII** | Store | 4^0^C |  |  |  |  |
