# Supplementary material for: Pyramiding of bacterial blight resistance genes into promising restorer BRRI31R line through marker-assisted backcross breeding and evaluation of agro-morphological and physiochemical characteristics of developed resistant restorer lines
Source: PLoS One. 2024 Jun 12;19(6):e0301342. doi: 10.1371/journal.pone.0301342 (PMC11168670; doi:10.1371/journal.pone.0301342)
Supplement: S2 Table — (DOCX) [file pone.0301342.s002.docx]

**S2 Table**. **List of trait-based SNP markers used to screen the pyramided restorer lines.**

| **Trait category** | **Trait** | **Target QTL** | **Customer**  **Marker ID** | **Intertek**  **SNP ID** | **Chromosome** | **Position** | **Favorable**  **allele** | **Unfavorable**  **allele** | **Allele source** |
| --- | --- | --- | --- | --- | --- | --- | --- | --- | --- |
| **Biotic stress** | Blast | Pb1 | Pb1_1 | snpOS00478 | 11 | 22862467 | T | C | Pokkali 26869 |
| **Biotic stress** | Blast | Pi9 | Pi9_1 | snpOS00451 | 6 | 10389610 | C | G | Pi9 (DQ285630) |
| **Biotic stress** | Blast | Pita | Pi-ta | snpOS00006 | 12 | 10607554 | C | A | - |
| **Biotic stress** | Blast | qPi33 | Pi33_1 | snpOS00468 | 8 | 6114723 | T | G | IR64 |
| **Biotic stress** | BLB | *xa13* | xa13_1 | snpOS00493 | 8 | 26727222 | C | G | IRBB60, some aus |
| **Biotic stress** | BLB | *Xa21* | *Xa21*_SKEP | snpOS00061 | 11 | 21273678 | C | G | IRBB60 |
| **Biotic stress** | BLB | *xa5* | *xa5*-S1_SKEP | snpOS00054 | 5 | 437499 | AG | TC | IRRI 154, FR13A |
| **Biotic stress** | Insect | BPH17 | Bph17_3 | snpOS00430 | 4 | 6957474 | G | A | Rathu Heenati - Liu *et al.,* 15 |
| **Biotic stress** | Insect | BPH32 | BPH32 | snpOS00442 | 6 | 1223332 | G | C | Honderawala,  Rc 222 |
| **Biotic stress** | Insect | qGm4(t) | Gm4_3 | snpOS00466 | 8 | 5466566 | A | G | Abhaya |
| **Biotic stress** | Insect | qGm4(t) | Gm4_4 | snpOS00467 | 8 | 5591019 | C | G | Abhaya |
| **Grain quality** | Grain quality | Waxy | Wx-A_group | snpOS00445 | 6 | 1768724 | C | T | Wx(a) - except Basmati |
| **Grain quality** | Grain quality | Waxy | Wx-A-NB | snpOS00446 | 6 | 1768795 | G | A | Wx(a) - Nona Bokra |
| **Grain quality** | Grain quality | Waxy | Wx-GBSS-ex10 | snpOS00038 | 6 | 1768998 | T | C | Wx(a)-Rc222, Exon 10 |
| **Grain quality** | Grain quality | Chalk5 | chalk5_576 | snpOS00024 | 5 | 3340295 | G | A | Minghui63 |
| **Yield components** | Grain yield | Gn1 | Gn1a_1 | snpOS00396 | 1 | 5276521 | T | A | Swarna (A8/AP9) allele |
| **Abiotic stress** | Salt | qNa1L | qSES1-2_2 | snpOS00409 | 1 | 39826525 | C | T | FL478, Capsule |
| **Abiotic stress** | Salt | qNa1L | qSES1-2_3 | snpOS00410 | 1 | 40362958 | A | G | FL478, Capsule |
| **Abiotic stress** | Salt | qNa1L | qSES1-2_4 | snpOS00411 | 1 | 40611906 | T | A | FL478, Capsule |
| **Abiotic stress** | Salt | Saltol | Saltol-Aus | snpOS00397 | 1 | 11460344 | T | G | FL478, IR 107321-1-141-3-120 |
