# Supplementary material for: Pyramiding of bacterial blight resistance genes into promising restorer BRRI31R line through marker-assisted backcross breeding and evaluation of agro-morphological and physiochemical characteristics of developed resistant restorer lines
Source: PLoS One. 2024 Jun 12;19(6):e0301342. doi: 10.1371/journal.pone.0301342 (PMC11168670; doi:10.1371/journal.pone.0301342)
Supplement: S3 Table — (DOCX) [file pone.0301342.s003.docx]

| **S3 Table. List of testcross F_1_s of pyramided lines.** | |
| --- | --- |
| **Sl. no.** | **Testcross F_1_s of pyramided lines** |
| **01** | IR75608B x BRRI31R-MASP1 |
| **02** | IR75608B x BRRI31R-MASP2 |
| **03** | IR75608B x BRRI31R-MASP3 |
| **04** | IR75608B x BRRI31R-MASP4 |
| **05** | IR75608B x BRRI31R-MASP5 |
| **06** | IR79156Ax BRRI31R-MASP1 |
| **07** | IR79156Ax BRRI31R-MASP2 |
| **08** | IR79156Ax BRRI31R-MASP3 |
| **09** | IR79156Ax BRRI31R-MASP4 |
| **10** | IR79156Ax BRRI31R-MASP5 |
