# Supplementary material for: Pyramiding of bacterial blight resistance genes into promising restorer BRRI31R line through marker-assisted backcross breeding and evaluation of agro-morphological and physiochemical characteristics of developed resistant restorer lines
Source: PLoS One. 2024 Jun 12;19(6):e0301342. doi: 10.1371/journal.pone.0301342 (PMC11168670; doi:10.1371/journal.pone.0301342)
Supplement: S1 Raw images — (DOCX) [file pone.0301342.s004.docx]

BC_3_F_4_ line

Artificial inoculation to evaluate disease spectrum

BC_3_F_3_

BRRI31R **×** IRBB60

MAS and phenotypic screening

Make a test cross between pyramided restorer lines and CMS lines for evaluation of restoring ability in restorer lines by conventional method

BC_1_F_1_ **×** BRRI31R

BC_2_F_1_ **×** BRRI31R

BC_3_F_1_

BC_3_F_2_ line

F_1_ **×** BRRI31R

Selection of homozygous bacterial blight resistant restorer (R) lines in the background of BRRI31R

BC_3_F_5_ line

Self

**Fig. 1.** Schematic representation of the introgression of *xa5, xa13* and *Xa21* into the restorer line


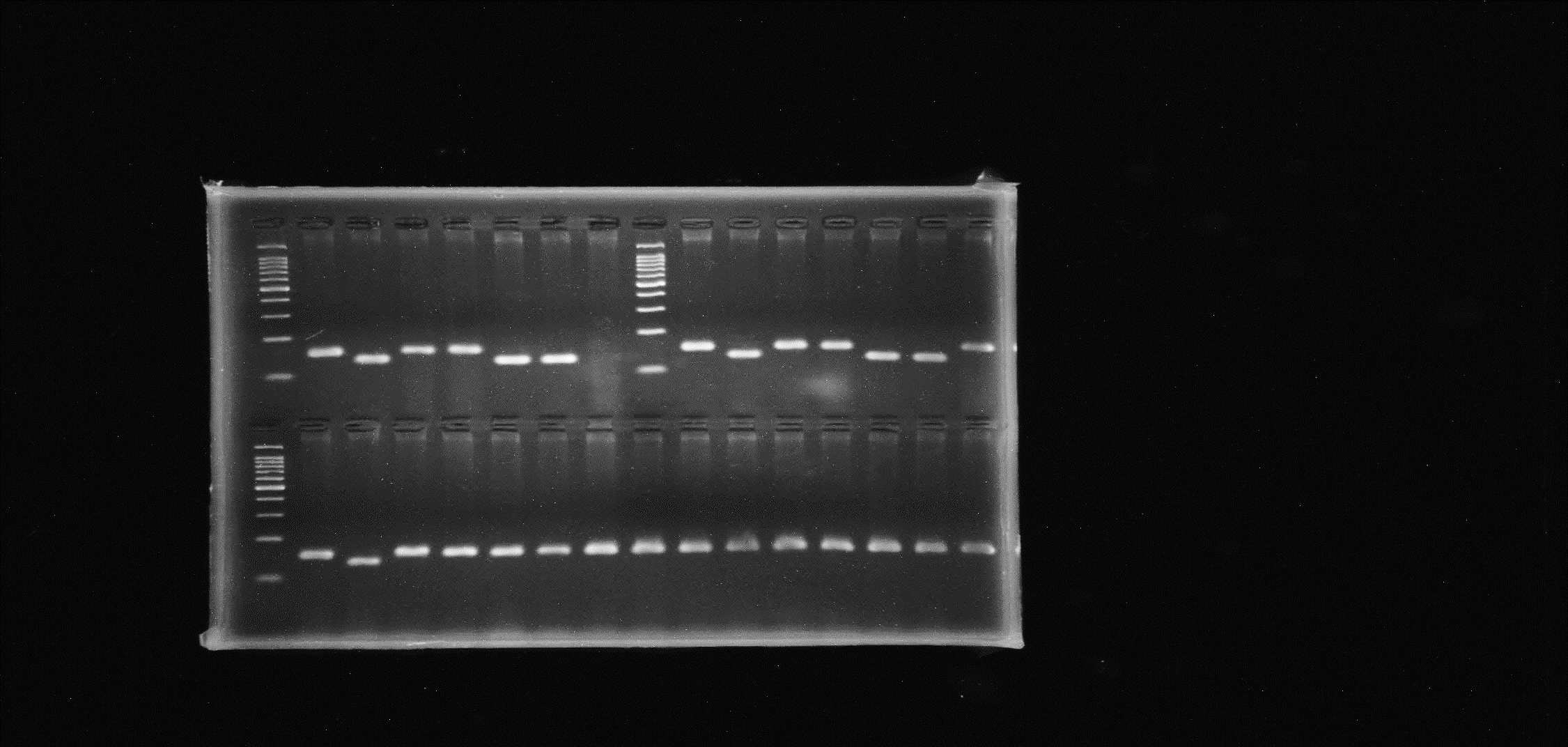

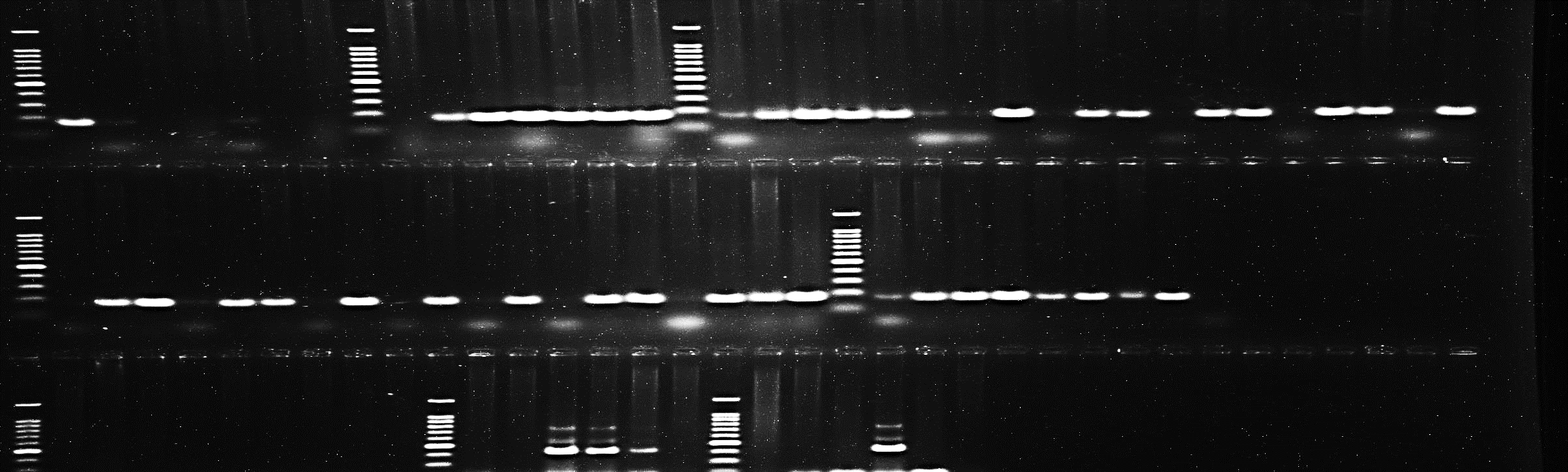

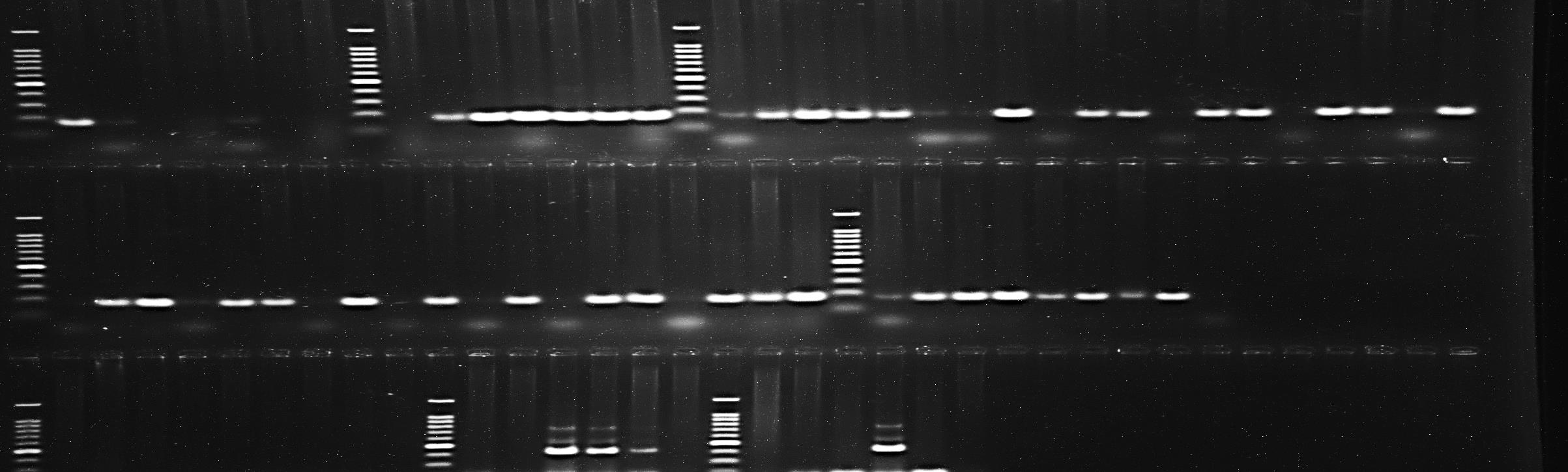


**100bpL**

**IRBB60 (DP)**

**IR24 (Sus.ck)**

**BRRI31R (RP)**

**100bpL**

**IRBB60 (DP)**

**IR24 (Sus.ck)**

**BRRI31R (RP)**

**100bpL**

**IRBB60 (DP)**

**IR24 (Sus.ck)**

**BRRI31R (RP)**

**1KbL**

**IRBB60 (DP)**

**IR24 (Sus.ck)**

**BRRI31R (RP)**


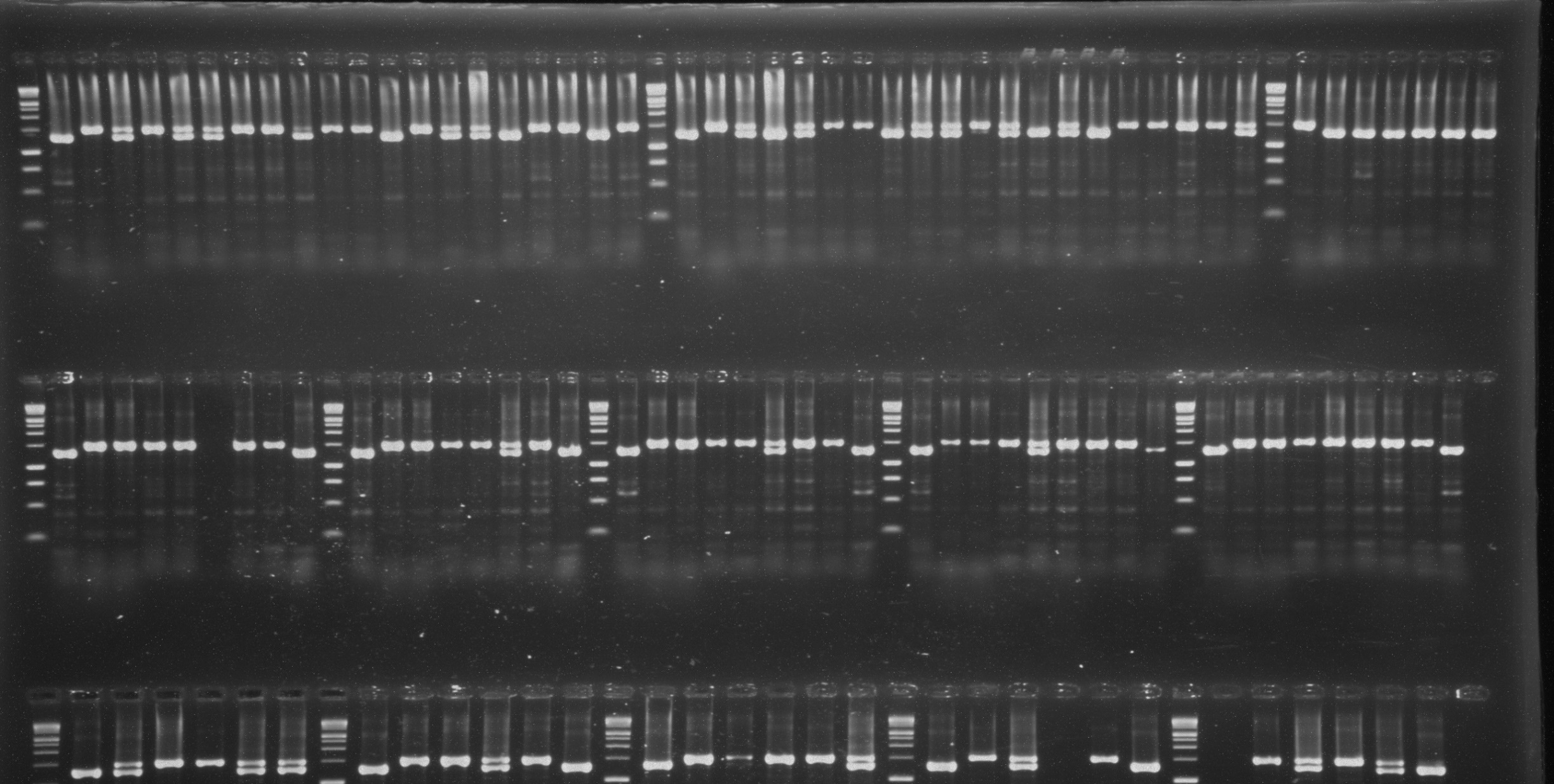


**1KbL**

**IRBB60 (DP)**

**IR24 (Sus.ck)**

**BRRI31R (RP)**


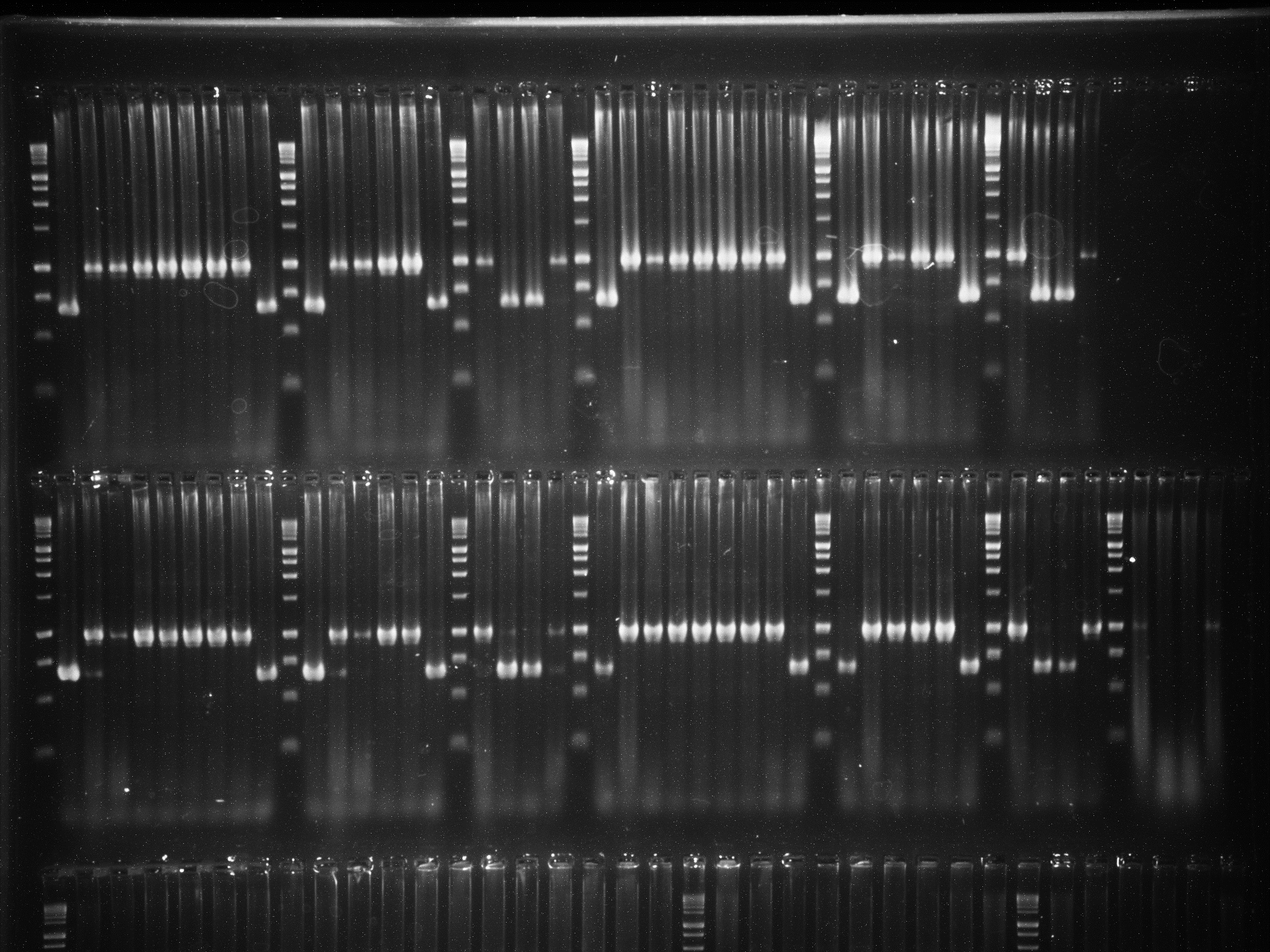


**S allele**

**R allele**

***Xa4***

***xa5***

***xa13***

***Xa21***

**Fig. 2a. Gel photographs showing banding pattern of the markers for the four bacterial blight(BB) resistant genes such as Xa4, xa5, xa13 and Xa21 in the parents**

**Fig. 2b.** Phenotypic screening of selected parents along with resistant and susceptible check against BXo races of bacterial blight (BB) disease.

BRRI31R

IRBB60 (Res.Ck)

IR24 (Sus.Ck)


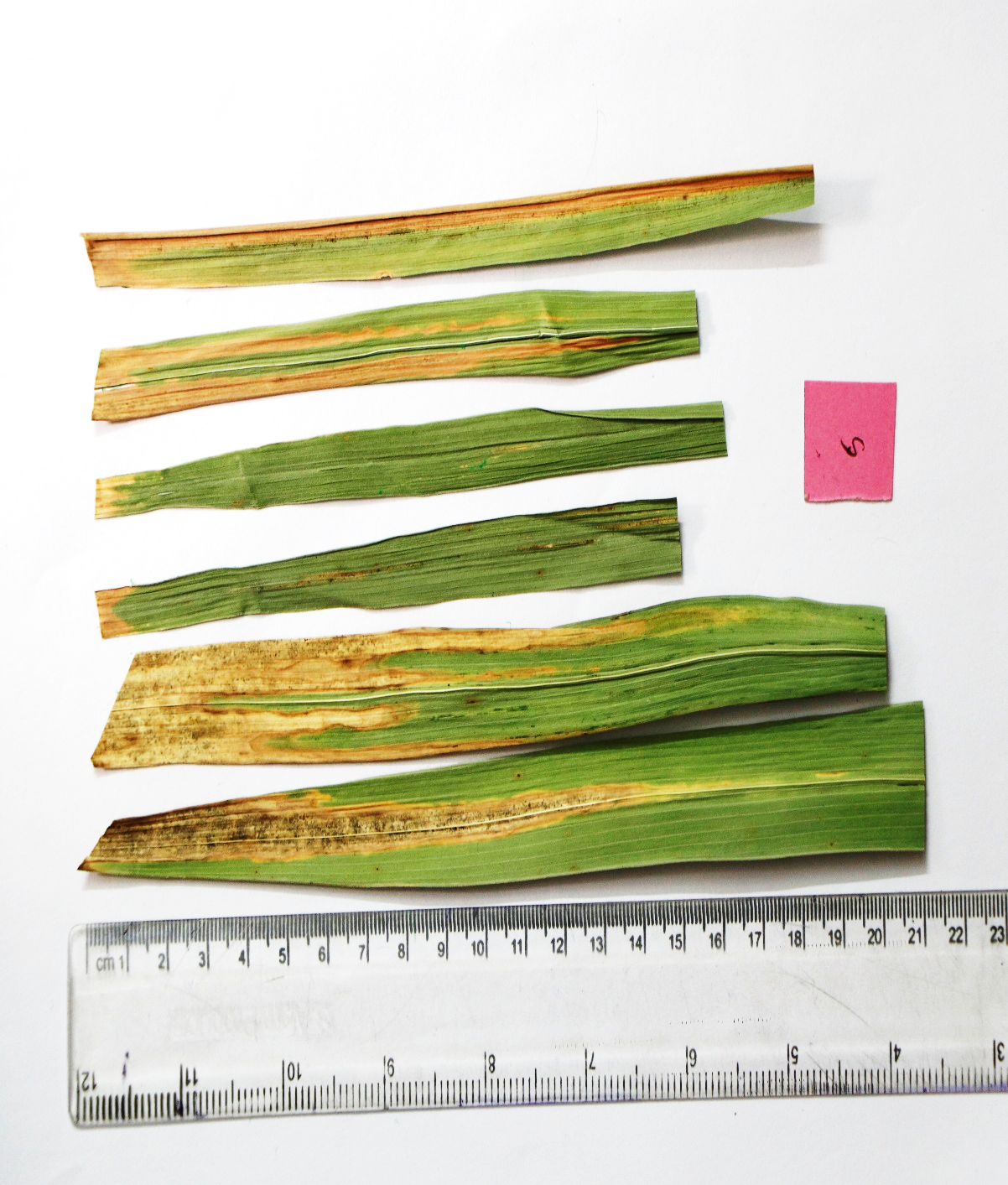


| **L1** | **P1** | **P4** | **P2** | **1** | **2** | **3** | **4** | **5** | **P3** |
| --- | --- | --- | --- | --- | --- | --- | --- | --- | --- |

| **L1** | **P1** | **P2** | **P5** | **1** | **2** | **3** | **4** | **5** | **P4** |
| --- | --- | --- | --- | --- | --- | --- | --- | --- | --- |


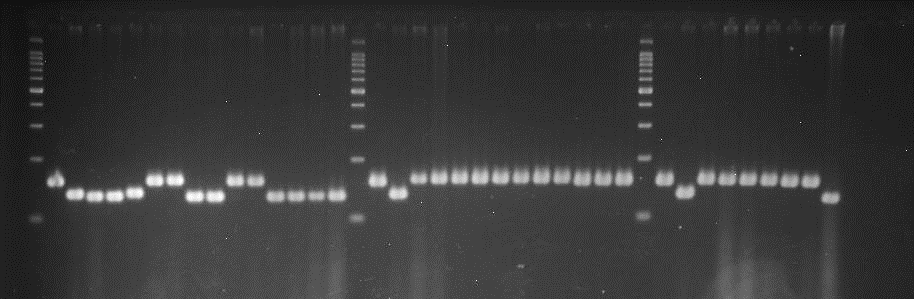


**(a) *Xa4***

**500**

**100**

**120**

**150**

**200**

**300**

**500**


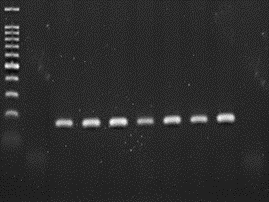


**100**

**198**

**300**

**(b) *xa5***

| **L2** | **P1** | **P2** | **P6** | **1** | **2** | **3** | **4** | **5** | **P4** |
| --- | --- | --- | --- | --- | --- | --- | --- | --- | --- |

| **L2** | **P1** | **P2** | **P7** | **1** | **2** | **3** | **4** | **5** | **P4** |
| --- | --- | --- | --- | --- | --- | --- | --- | --- | --- |


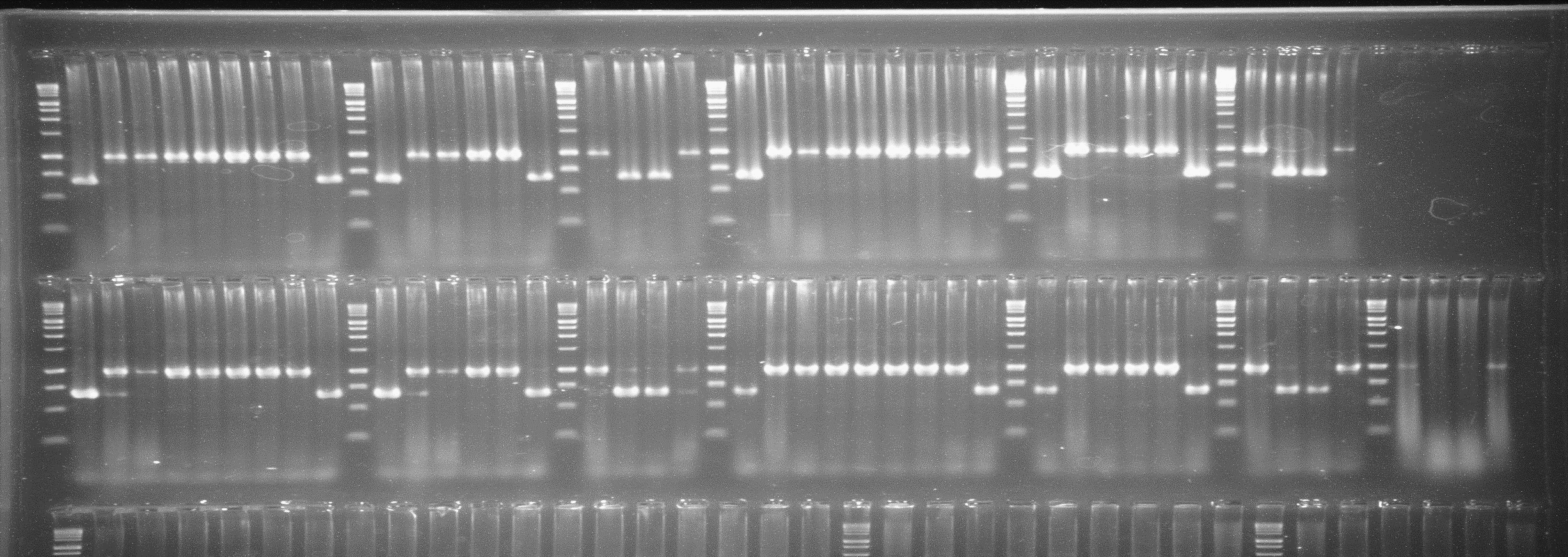


**1000bp**

**1500bp**

**650bp**

**250bp**

**500bp**

**(d) *Xa21***


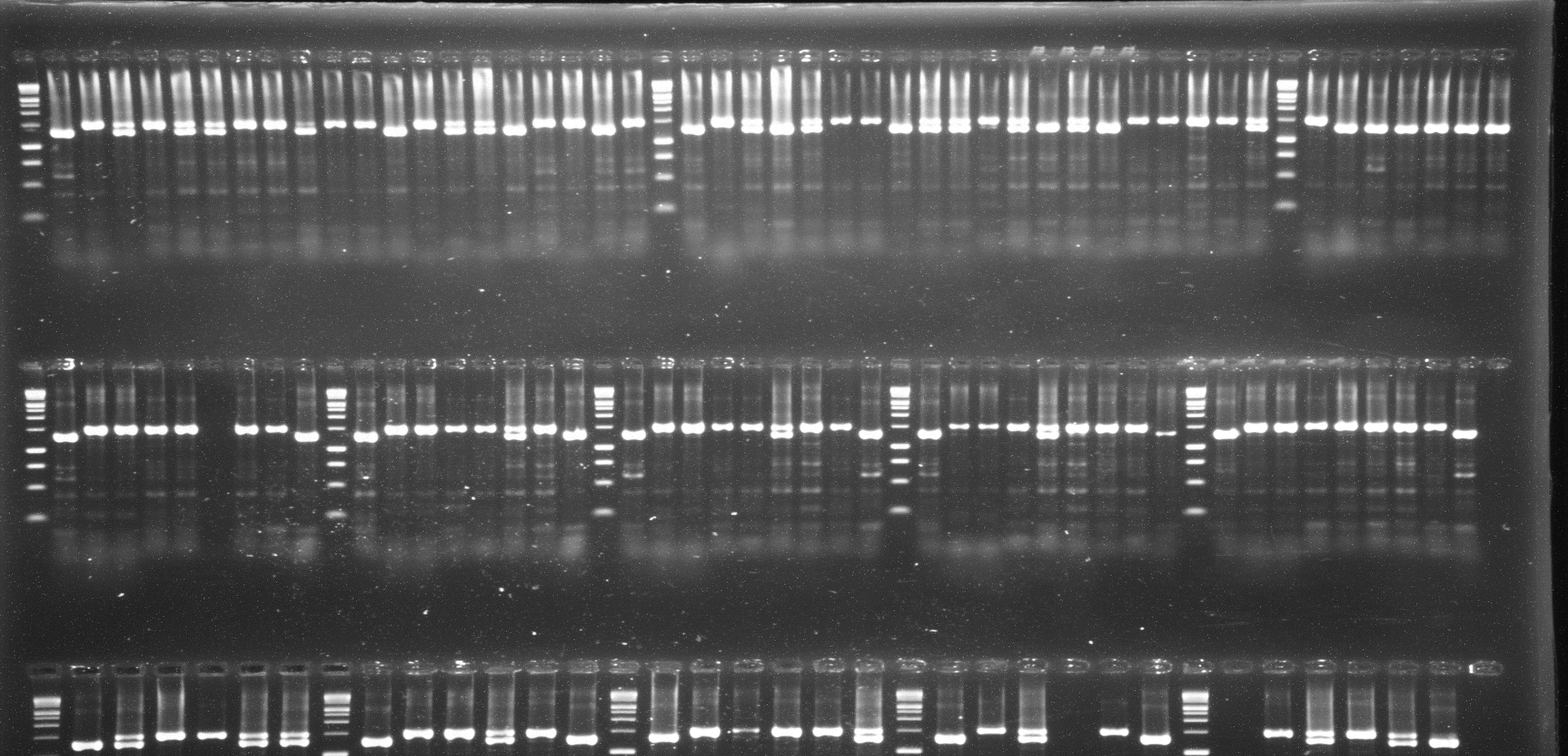


**1322bp**

**1522bp**

**250bp**

**500bp**

**(c) *xa13***

| **L3** | **P8** | **P9** | **1** | **2** | **3** | **4** | **5** |
| --- | --- | --- | --- | --- | --- | --- | --- |

| **L1** | **P8** | **P9** | **1** | **2** | **3** | **4** | **5** |
| --- | --- | --- | --- | --- | --- | --- | --- |


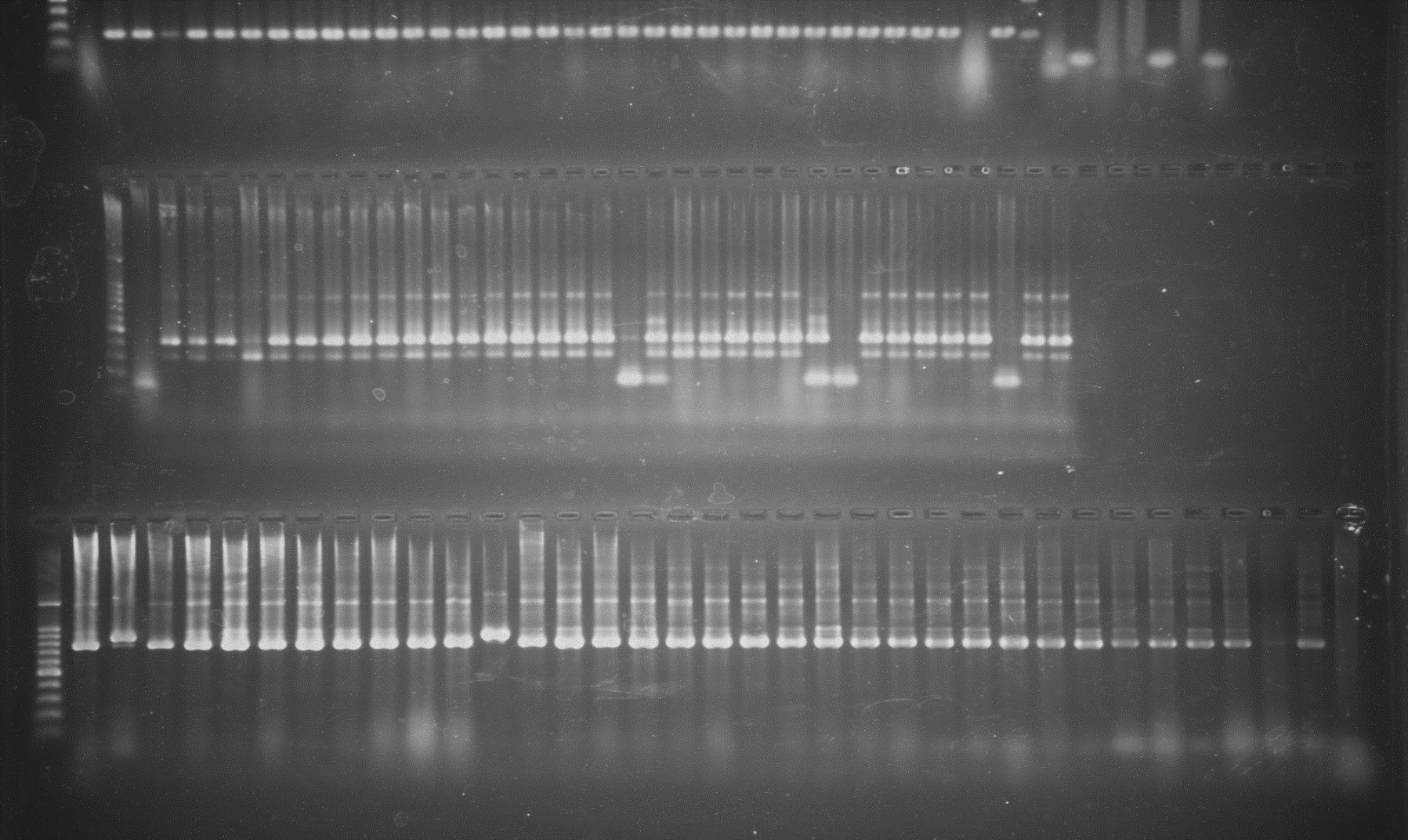


**115bp R**

**85bp NR**


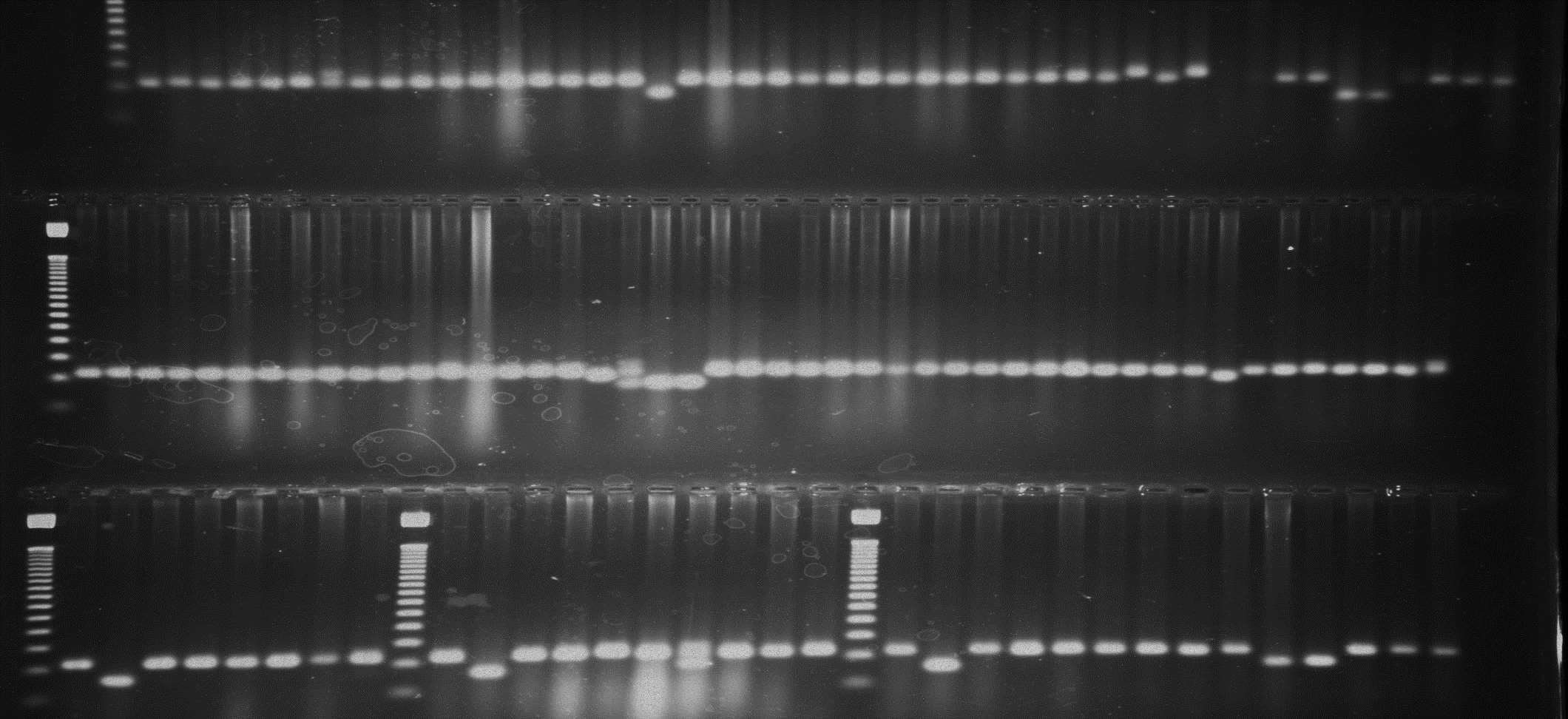


**(e) *Rf3***

**50bp**

**900bp NR**

**100bp**

**(f) *Rf4***

**800bp R**

**Fig. 3.** Gel image of *Xa4*, *xa5*, *xa13*, *Xa21*, *Rf3* and *Rf4* genes in fixed restorer lines of BC_3_F_5._ Here, L1, 100bp; L2, 1kb; L3, 50bp; P1, BRRI31R (Recipient Parent); P2, IRBB60 (Donor Parent); P3, IRBB4 (resistant check for *Xa4* gene); P4, IR24 (susceptible check); P5, IRBB5 (resistant check for *xa5* gene); P6, IRBB13 (resistant check for *xa13* gene); P7, IRBB21 (resistant check for *Xa21* gene); P8, IR96479-81-7-1-1-B-1-1-1-R (RA, restorer allele); P9, IR58025A (NR, non-restorer allele).


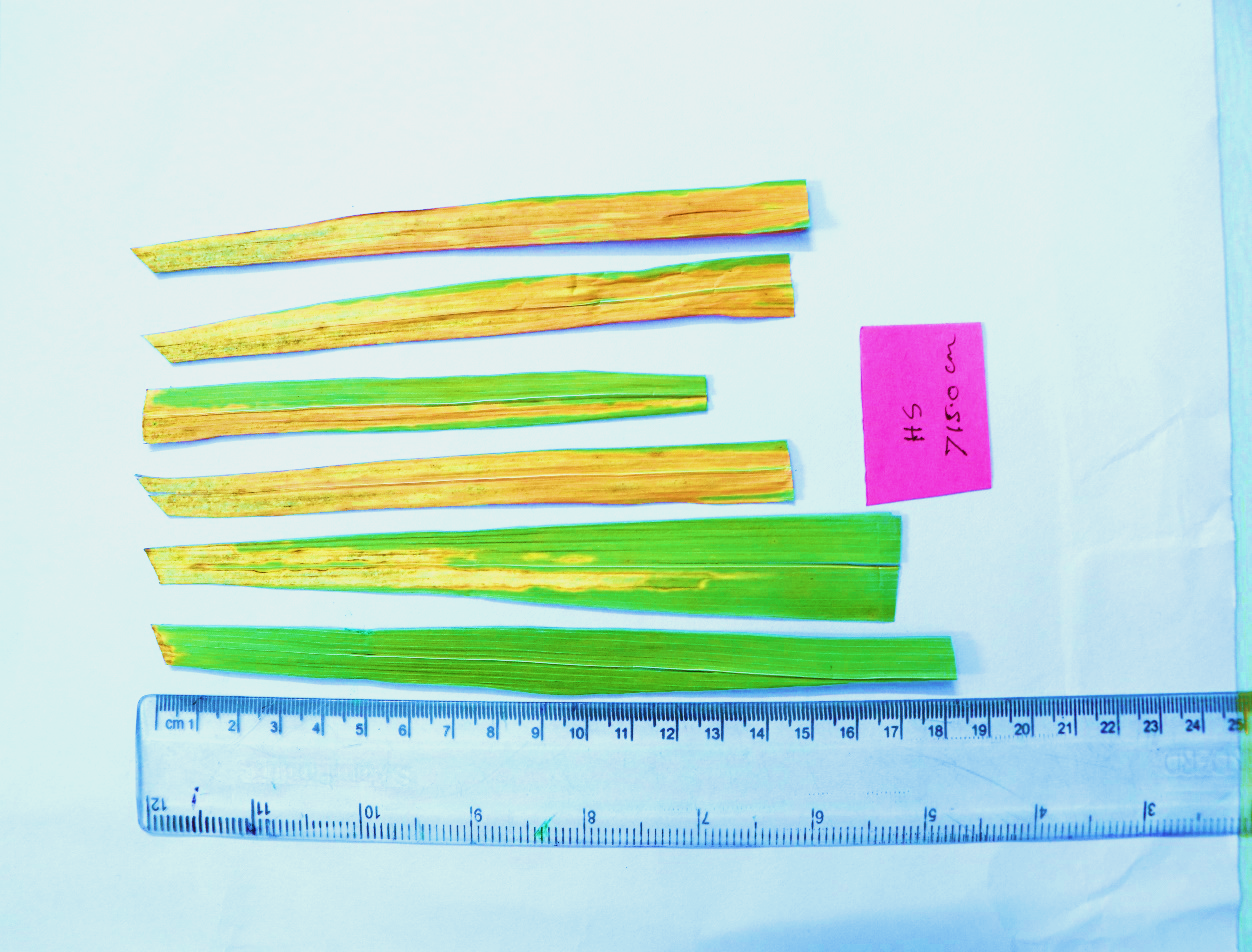

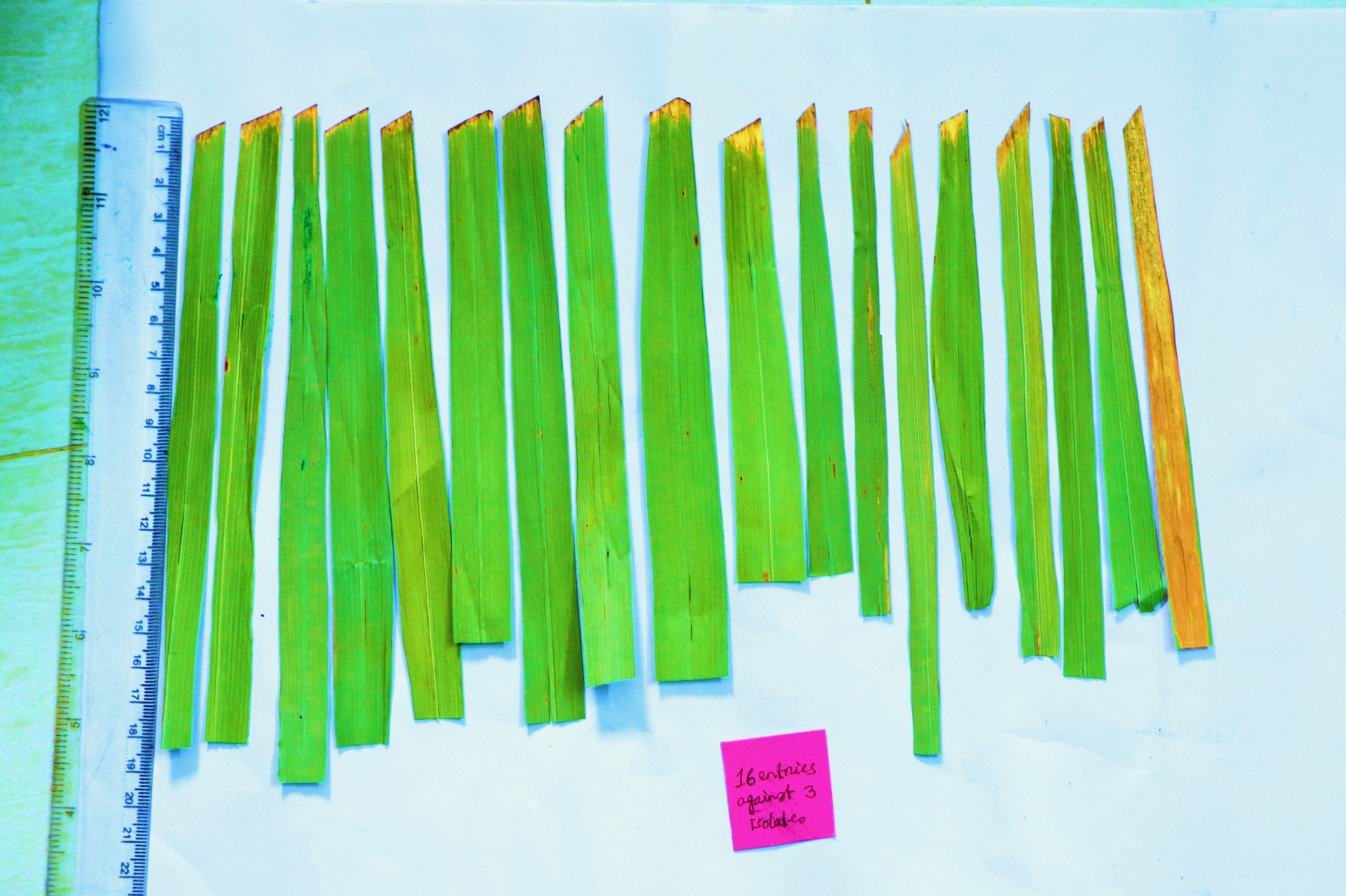


**BRRI31R-MASP1**

**BRRI31R-MASP2**

**BRRI31R-MASP3**

**BRRI31R-MASP4**

**BRRI31R-MASP5**

**BRRI31R-RP**

**IRBB60-DP**

**IR24 – Sus.Ck**

**Figure 4.** Phenotypic screening of selected pyramiding restorer lines along with respective recurrent parent, donor parent and susceptible check against BXo races of bacterial blight (BB) disease.


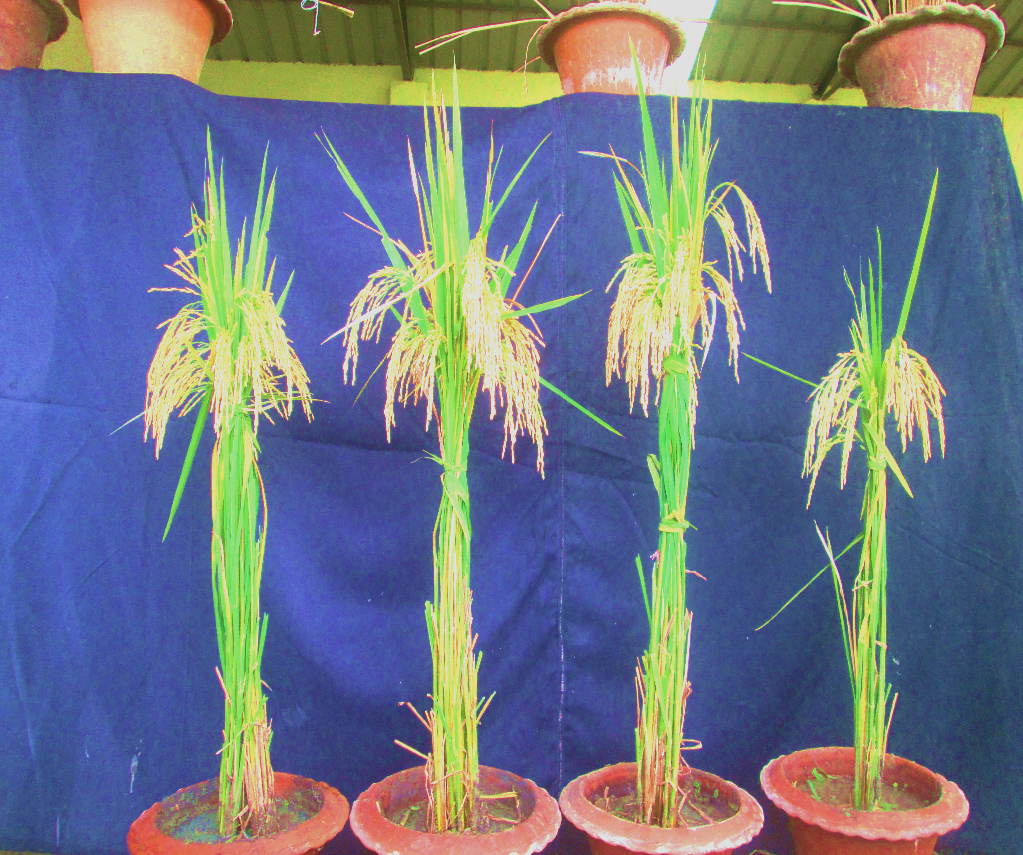

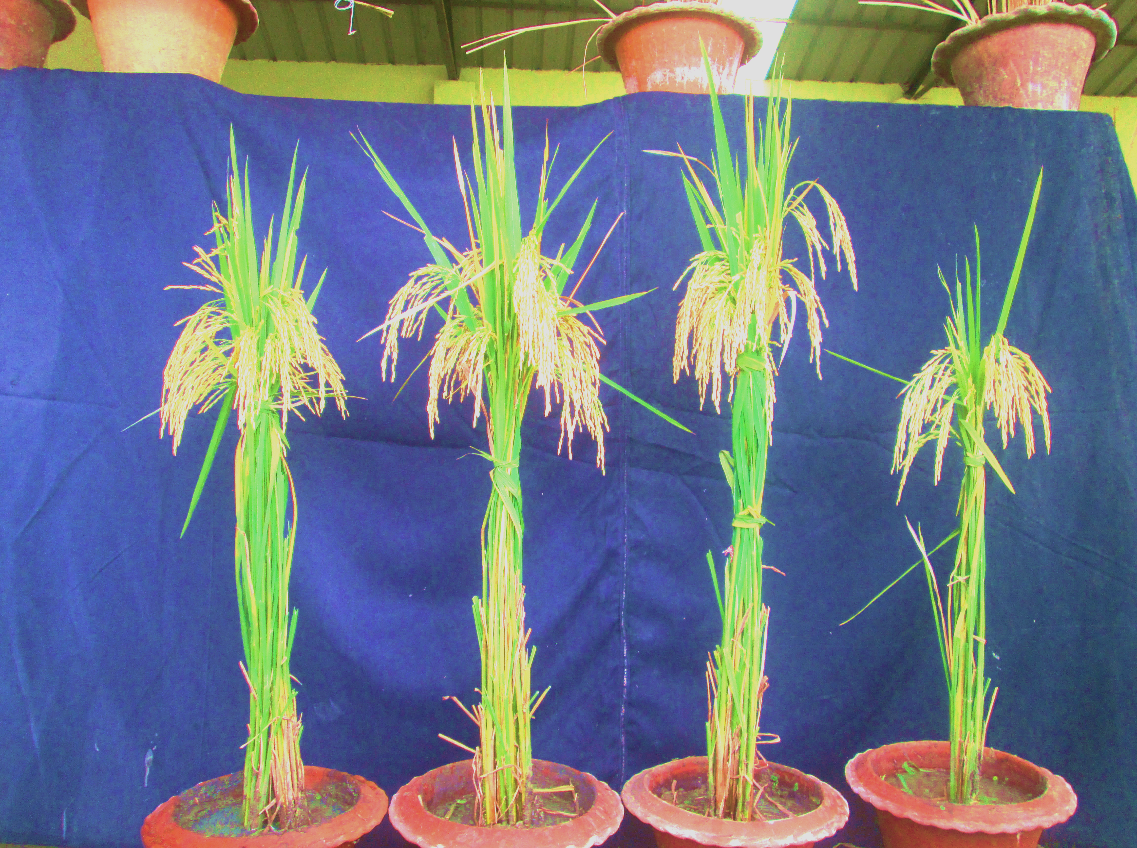

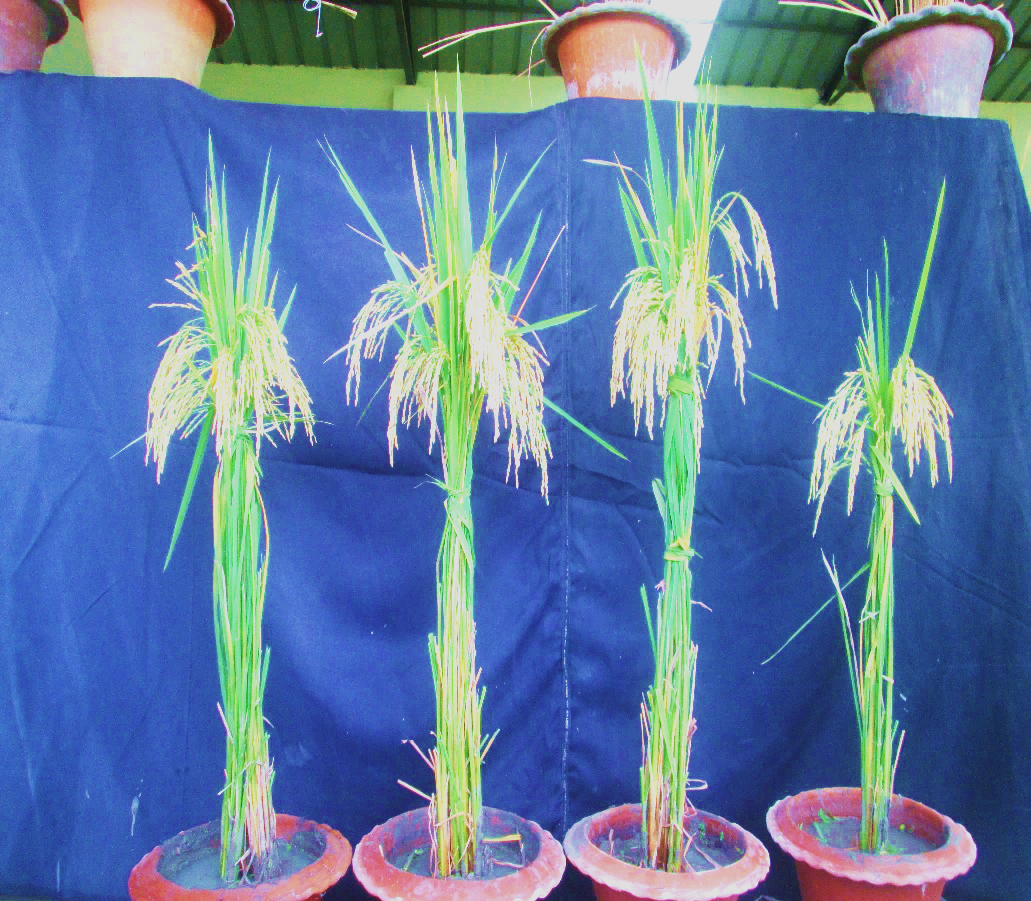

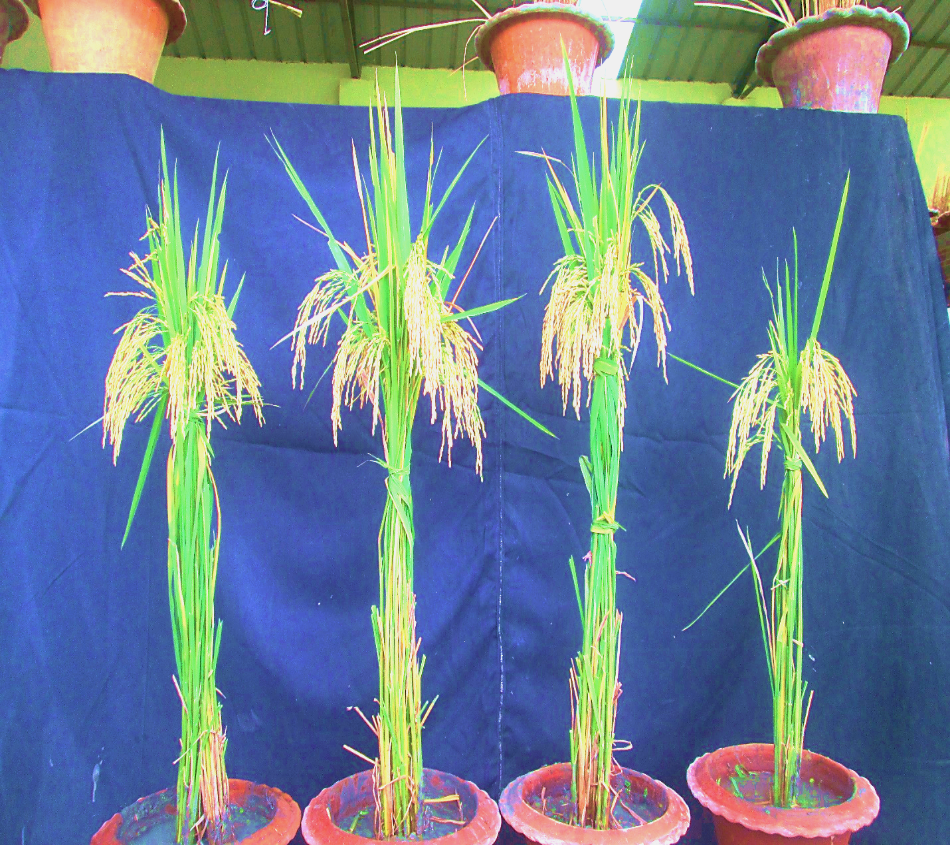

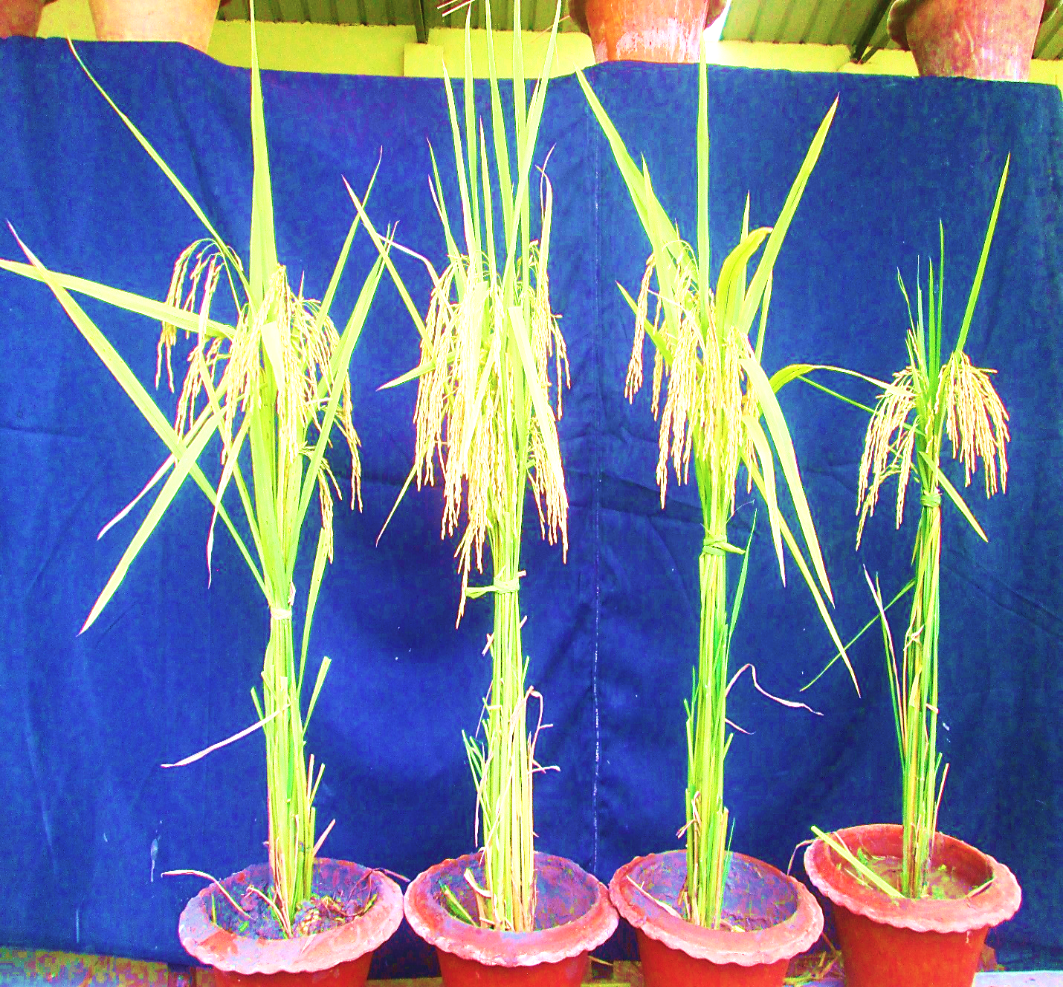

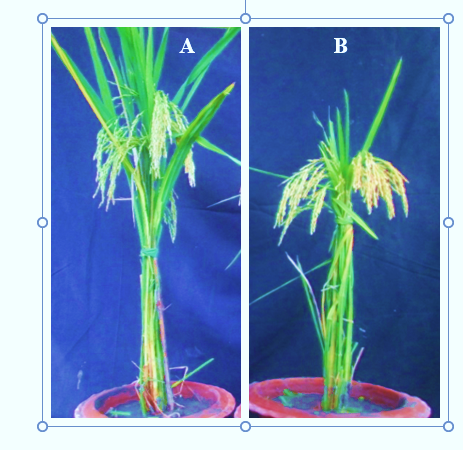


**BRRI31R-RP**

**BRRI31R-MASP1**

**BRRI31R-MASP2**

**BRRI31R-MASP3**

**BRRI31R-MASP4**

**BRRI31R-MASP5**

**Figure 5.** Phenotype of the selected four gene pyramided restorer lines in BC_3_F_5_ progenies compared with recurrent parent BRRI31R.
