# Supplementary material for: Pyramiding of bacterial blight resistance genes into promising restorer BRRI31R line through marker-assisted backcross breeding and evaluation of agro-morphological and physiochemical characteristics of developed resistant restorer lines
Source: PLoS One. 2024 Jun 12;19(6):e0301342. doi: 10.1371/journal.pone.0301342 (PMC11168670; doi:10.1371/journal.pone.0301342)
Supplement: Attachment — Submitted filename: PONE-D-23-34671_reviewer.pdf [file pone.0301342.s006.pdf]

PLOS ONE

Pyramiding of bacterial blight resistance genes into promising restorer BRRI31R line through marker-assisted backcross breeding and evaluation of agro-morphological and physiochemical characteristics of developed resistant restorer lines

--Manuscript Draft--

Manuscript Number:	PONE-D-23-34671
Article Type:	Research Article
Full Title:	Pyramiding of bacterial blight resistance genes into promising restorer BRRI31R line through marker-assisted backcross breeding and evaluation of agro-morphological and physiochemical characteristics of developed resistant restorer lines
Short Title:	Development of bacterial blight resistance restorer lines through marker-assisted selection breeding.
Corresponding Author:	M. A. Latif<br>BRRI: Bangladesh Rice Research Institute<br>Gazipur, Joydebpur BANGLADESH
Keywords:	Bacterial blight, Marker-assisted backcrossing, Pyramiding, Restorer line.
Abstract:	BRRI31R is one of Bangladesh's most promising restorer lines due to its abundant pollen producing capacity, strong restoring ability, good combining ability, high outcrossing rate and genetically diverse from CMS line, but highly susceptible to bacterial blight (BB) disease of rice. The present study highlighted the pyramiding of effective BB resistance genes into the background of BRRI31R, by incorporating three major BB resistance genes, xa5, xa13 and Xa21 through marker–assisted backcrossing (MABC). Whole backcross progenies were confirmed and advanced based on foreground selection associated with the genes. Pyramided lines were used for pathogenicity test against five BXo races (BXo93, BXo220, BXo822, BXo826, BXo887) and confirmed the dominant fertility restore genes, Rf3 and Rf4 and further validated against SNP markers for more confirmation of target resistance genes. All pyramided restorer lines consisted of Xa4 (in built), xa5, xa13, Xa21, and Chalk5 with two fertility restorer genes, Rf3, Rf4.Among them, two pyramid restorer lines, BRRI31R-MASP3 and BRRI31R-MASP4 contained additional blast resistance gene Pita and these lines exhibited high levels of resistance against five virulent BXo races and were very similar to respective recurrent parent. Pyramided restorer lines showed intermediate amylose content (<25%). So, gene pyramided restorer lines, BRRI31R-MASP3 and BRRI31R-MASP4 can directly be used as a male parent for the development of new BB resistant hybrid rice variety and also replacement as a restorer line of BRRI hybrid dhan5 and 7 to enhance the quality of hybrid seeds as well as rice production in Bangladesh.
Order of Authors:	Anowara Akter
	Lutful Hassan
	Sheikh Arafat Islam Nihad
	Md. Jamil Hasan
	Arif Hasan Khan Robin
	Mahmuda Khatun
	Anika Tabassum
	M. A. Latif
Additional Information:	
Question	Response
Financial Disclosure<br><br>Enter a financial disclosure statement that	Yes

Powered by Editorial Manager® and ProduXion Manager® from Aries Systems Corporation

describes the sources of funding for the work included in this submission. Review the submission guidelines for detailed requirements. View published research articles from *PLOS ONE* for specific examples.<br><br>This statement is required for submission and **will appear in the published article** if the submission is accepted. Please make sure it is accurate.<br><br>**Funded studies**<br>Enter a statement with the following details:<br>• Initials of the authors who received each award<br>• Grant numbers awarded to each author<br>• The full name of each funder<br>• URL of each funder website<br>• Did the sponsors or funders play any role in the study design, data collection and analysis, decision to publish, or preparation of the manuscript?<br><br>Did you receive funding for this work?	
Please add funding details.<br>as follow-up to "**Financial Disclosure**<br><br>Enter a financial disclosure statement that describes the sources of funding for the work included in this submission. Review the submission guidelines for detailed requirements. View published research articles from *PLOS ONE* for specific examples.<br><br>This statement is required for submission and **will appear in the published article** if the submission is accepted. Please make sure it is accurate.	Authors received funds from Strengthening Physical Infrastructure and Research Activities (SPIRA) Project, Bangladesh Rice Research Institute, through the Ministry of Agriculture, Bangladesh to conduct this experiment.

**Funded studies**<br>Enter a statement with the following details:<br>• Initials of the authors who received each award<br>• Grant numbers awarded to each author<br>• The full name of each funder<br>• URL of each funder website<br>• Did the sponsors or funders play any role in the study design, data collection and analysis, decision to publish, or preparation of the manuscript?<br><br>Did you receive funding for this work?"	
Please select the country of your main research funder (please select carefully as in some cases this is used in fee calculation).<br>as follow-up to "**Financial Disclosure**<br><br>Enter a financial disclosure statement that describes the sources of funding for the work included in this submission. Review the submission guidelines for detailed requirements. View published research articles from *PLOS ONE* for specific examples.<br><br>This statement is required for submission and **will appear in the published article** if the submission is accepted. Please make sure it is accurate.<br><br>**Funded studies**<br>Enter a statement with the following details:<br>• Initials of the authors who received each award<br>• Grant numbers awarded to each author<br>• The full name of each funder<br>• URL of each funder website<br>• Did the sponsors or funders play any role in the study design, data collection and analysis, decision to publish, or preparation of the manuscript?<br><br>Did you receive funding for this work?"	BANGLADESH - BD
**Competing Interests**	The authors have declared that no competing interests exist.

Use the instructions below to enter a competing interest statement for this submission. On behalf of all authors, disclose any competing interests that could be perceived to bias this work—acknowledging all financial support and any other relevant financial or non-financial competing interests.<br><br>This statement is required for submission and **will appear in the published article** if the submission is accepted. Please make sure it is accurate and that any funding sources listed in your Funding Information later in the submission form are also declared in your Financial Disclosure statement.<br><br>View published research articles from *PLOS ONE* for specific examples.<br><br>**NO authors have competing interests**<br><br>Enter: *The authors have declared that no competing interests exist.*<br><br>**Authors with competing interests**<br><br>Enter competing interest details beginning with this statement:<br><br>*I have read the journal's policy and the authors of this manuscript have the following competing interests: [insert competing interests here]*<br><br>* typeset	
**Ethics Statement**<br><br>Enter an ethics statement for this submission. This statement is required if the study involved:<br><br>• Human participants<br>• Human specimens or tissue<br>• Vertebrate animals or cephalopods<br>• Vertebrate embryos or tissues	N/A

Powered by Editorial Manager® and ProduXion Manager® from Aries Systems Corporation

• Field research<br><br>Write "N/A" if the submission does not require an ethics statement.<br><br>General guidance is provided below. Consult the submission guidelines for detailed instructions. **Make sure that all information entered here is included in the Methods section of the manuscript.**	

Powered by Editorial Manager® and ProduXion Manager® from Aries Systems Corporation

**Format for specific study types**<br><br>**Human Subject Research (involving human participants and/or tissue)**<br>• Give the name of the institutional review board or ethics committee that approved the study<br>• Include the approval number and/or a statement indicating approval of this research<br>• Indicate the form of consent obtained (written/oral) or the reason that consent was not obtained (e.g. the data were analyzed anonymously)<br><br>**Animal Research (involving vertebrate animals, embryos or tissues)**<br>• Provide the name of the Institutional Animal Care and Use Committee (IACUC) or other relevant ethics board that reviewed the study protocol, and indicate whether they approved this research or granted a formal waiver of ethical approval<br>• Include an approval number if one was obtained<br>• If the study involved *non-human primates*, add *additional details* about animal welfare and steps taken to ameliorate suffering<br>• If anesthesia, euthanasia, or any kind of animal sacrifice is part of the study, include briefly which substances and/or methods were applied<br><br>**Field Research**<br><br>Include the following details if this study involves the collection of plant, animal, or other materials from a natural setting:<br>• Field permit number<br>• Name of the institution or relevant body that granted permission	
**Data Availability**<br><br>Authors are required to make all data underlying the findings described fully available, without restriction, and from the time of publication. PLOS allows rare exceptions to address legal and ethical concerns. See the PLOS Data Policy and FAQ for detailed information.	Yes - all data are fully available without restriction

A Data Availability Statement describing where the data can be found is required at submission. Your answers to this question constitute the Data Availability Statement and **will be published in the article**, if accepted.<br><br>**Important:** Stating 'data available on request from the author' is not sufficient. If your data are only available upon request, select 'No' for the first question and explain your exceptional situation in the text box.<br><br>Do the authors confirm that all data underlying the findings described in their manuscript are fully available without restriction?	
**Describe where the data may be found in full sentences. If you are copying our sample text, replace any instances of XXX with the appropriate details.**<br><br>• If the data are **held or will be held in a public repository**, include URLs, accession numbers or DOIs. If this information will only be available after acceptance, indicate this by ticking the box below. For example: *All XXX files are available from the XXX database (accession number(s) XXX, XXX.).*<br>• If the data are all contained **within the manuscript and/or Supporting Information files**, enter the following: *All relevant data are within the manuscript and its Supporting Information files.*<br>• If neither of these applies but you are able to provide **details of access elsewhere**, with or without limitations, please do so. For example:<br><br>*Data cannot be shared publicly because of [XXX]. Data are available from the XXX Institutional Data Access / Ethics Committee (contact via XXX) for researchers who meet the criteria for access to confidential data.*<br><br>*The data underlying the results presented in the study are available from (include the name of the third party*	All relevant data are within the manuscript and its Supporting Information files.

and contact information or URL).<br>• This text is appropriate if the data are owned by a third party and authors do not have permission to share the data.<br><br>* typeset	
Additional data availability information:	Tick here if the URLs/accession numbers/DOIs will be available only after acceptance of the manuscript for publication so that we can ensure their inclusion before publication.

Powered by Editorial Manager® and ProduXion Manager® from Aries Systems Corporation

Manuscript Click here to access/download;Manuscript;Anowra et al., 2023.docx

Pyramiding of bacterial blight resistance genes into promising restorer BRRI31R line through marker-assisted backcross breeding and evaluation of agro-morphological and physiochemical characteristics of developed resistant restorer lines

Short title: Development of bacterial blight resistance restorer lines through marker-assisted selection breeding.

Anowara Akter[1*], Lutful Hassan[2], Sheikh Arafat Islam Nihad[3], Md. Jamil Hasan[1], Arif Hasan Khan Robin[2], Mahmuda Khatun[4], Anika Tabassum[5], Mohammad Abdul Latif*[1]

[1]Hybrid Rice Division, Bangladesh Rice Research Institute, Gazipur-1701, Bangladesh
[2]Genetics and Plant Breeding, Bangladesh Agricultural University, Mymensingh-2202, Bangladesh
[3] Plant Pathology Division, Bangladesh Rice Research Institute, Gazipur-1701, Bangladesh
[4]Genetics and Plant Breeding Division, Bangladesh Rice Research Institute, Gazipur-1701, Bangladesh
[5]Plant Breeding Division, Bangladesh Agricultural Research Institute (BARI), Gazipur-1701, Bangladesh

***Corresponding author:**

E-mail:
1. alatif1965@yahoo.com
2. anowaraa@yahoo.com

Abstract

BRRI31R is one of Bangladesh's most promising restorer lines due to its abundant pollen producing capacity, strong restoring ability, good combining ability, high outcrossing rate and genetically diverse from CMS line, but highly susceptible to bacterial blight (BB) disease of rice. The present study highlighted the pyramiding of effective BB resistance genes into the background of BRRI31R, by incorporating three major BB resistance genes, *xa5, xa13* and *Xa21* through marker–assisted backcrossing (MABC). Whole backcross progenies were confirmed and advanced based on foreground selection associated with the genes. Pyramided lines were used for pathogenicity test against five *BXo* races (*BXo*93, *BXo*220, *BXo*822, *BXo*826, *BXo*887) and confirmed the dominant fertility restore genes, *Rf3* and *Rf4* and further validated against SNP markers for more confirmation of target resistance genes. All pyramided restorer lines consisted of *Xa4* (in built), *xa5, xa13, Xa21*, and *Chalk5* with two fertility restorer genes, *Rf3, Rf4.* Among them, two pyramid restorer lines, BRRI31R-MASP3 and BRRI31R-MASP4 contained additional blast resistance gene *Pita* and these lines exhibited high levels of resistance against five virulent *BXo* races and were very similar to respective recurrent parent. Pyramided restorer lines showed intermediate amylose content (<25%). So, gene pyramided restorer lines, BRRI31R-MASP3 and BRRI31R-MASP4 can directly be used as a male parent for the development of new BB resistant hybrid rice variety and also replacement as a restorer line of BRRI hybrid dhan5 and 7 to enhance the quality of hybrid seeds as well as rice production in Bangladesh.

Keywords: Bacterial blight, Marker-assisted backcrossing, Pyramiding, Restorer line.

Introduction

Rice (*Oryza sativa* L.) is the world's most influential and economically significant crop, with more than half of the world's population relying on rice to meet their daily calorie requirements [1,2]. Bangladesh has secured the third position for total rice production in the world and food security depends on rice security [1,2]. It has been assumed that, in 2050, the population of Bangladesh will be 215.4 million, when 44.6 MT of clean rice will be required [3]. Climate change and the emergence of different biotic and abiotic stresses are threatening rice production in the world including Bangladesh [1,4,5]. To ensure the basic food demand of the burgeoning population of Bangladesh, hybrid rice could be a viable solution to increase rice production with a 15-20% yield advantage compared to inbred varieties [6,7].

In Bangladesh, till now, 32 rice diseases have been reported and based on severity and incidence, blast [8], sheath blight [9], BB [10,11], tungro [12] and false smut [13] considered as major disease across the country. At present, BB is one of the major threats to both inbred and hybrid rice cultivation in the world specifically in South-east Asia including Bangladesh [14–18]. In South and Southeast Asian nations, BB caused by *Xanthomonas oryzae* pv. *oryzae* (*Xoo*) causes 10-50% yield loss, but it can exceed 80%, depending on variety, crop stage, location, and favorable environment [19–21]. The input requirement of hybrid is more compared to inbred varieties and these phenomena favor the epidemics of diverse diseases and pests. High-yielding varieties require high dose of nitrogen which favors the occurrence of BB in different countries of the world including Bangladesh [22]. Ansari et al. [22] found that due to BB around 5.8-30.4% yield loss occurred in field conditions of Bangladesh. However, the damage caused by bacterial blight disease depends on many factors including location, virulence of the race, crop stage, environmental condition and cultivar [23,24]. But till now, no effective chemical control measure is suggested to combat the disease. So far, 237 hybrid rice varieties have been released in Bangladesh but no variety has any resistance characteristics because all hybrid parental lines are susceptible to available races of *Xoo* [25].

Therefore, stacking resistance genes into hybrid parental lines is a durable strategy to manage bacterial blight disease [25]. Moreover, by adopting conventional breeding it is tough to introgress more than one gene into the single lines and sometimes dominance and epistasis effects of genes make it more difficult to identify the effective gene [26,27]. However, the development of DNA markers linked to resistance genes makes it easy to detect two or more genes in the introgressed lines [8,28–30]. Trait-specific gene transformation through marker-assisted selection (MAS) is a very popular technique to develop inbred and hybrid lines with

desired characters [8,31]. To date, more than 46 resistance genes have been identified as effective against bacterial blight pathogens [32]. Among them, *Xa4*, *xa5*, *Xa7*, *xa13*, *Xa21*, and *Xa23* gens have been extensively deployed and incorporated into rice cultivars for the development of BB resistant cultivars [33]. Resistance genes i.e., *xa5*, *xa13* and *Xa21* were found effective for the bacterial blight pathogens of Bangladesh [23,25,34,35]. Moreover, molecular markers of these resistance genes are widely used in breeding programs to develop bacterial blight resistant rice varieties [36,37]. Moreover, it is crucial to characterize genotypes using trait-specific SNPs in order to examine the frequency of advantageous alleles for various traits of interest associated with biotic and abiotic stressors as well as grain quality characteristics. The high throughput SNP platform has made it possible to choose genotypes for elite parents with exceptional traits utilizing contemporary breeding techniques like marker-assisted forward breeding and genomic selection.

As a result of the aforementioned facts, the current study project was initiated with the following goals in mind: (i) introgression of *xa5, xa13 & Xa21* genes in the promising restorer lines, BRRI31R using MABC; (ii) assessment of introgressed pyramided restorer lines by molecular and conventional approach; (iii) confirmation of fertility restorer genes (iv) evaluation of morphological and grain quality characters of developed pyramided lines in comparison with respective recurrent parent. However, the development of pyramided BB resistant restorer line carrying four resistance genes (*Xa4*, *xa5, xa13* and *Xa21*) has a promising future to develop a bacterial blight-resistant hybrid variety in Bangladesh.

Materials and methods

Plant materials

BRRI31R is a restorer background with medium long slender, medium duration, strong restorers but susceptible to bacterial blight disease. IRBB60, a pyramided line carrying four resistance genes (*Xa4 + xa5 + xa13+ Xa21*) was used as the donor for introgression of BB resistance genes into the most promising restorer line BRRI31R. We considered this line as promising because this parental line was also used in the background of two popular hybrid rice varieties (BRRI hybrid dhan5 and BRRI hybrid dhan7) of Bangladesh. Before hybridization, the selected parents were used for morphological screening against five *BXo* races and validation of markers against BB resistance genes, *Xa4*, *xa5*, *xa13* and *Xa21* for foreground selection.

Marker-assisted breeding strategy for restorer line development

A cross was made between BRRI31R and IRBB60. The F_1 plants were backcrossed thrice with recurrent parent (BRRI31R) to produce BC_3F_1 plants, which were then selfed to get up to BC_3F_5 progenies. Whole backcross was confirmed and advanced from BC_3F_2 to BC_3F_5, based on gene-linked molecular markers. F_1 to BC_3F_1 were followed for background recovery of recurrent parent and foreground selection was started from BC_1F_1 generation and continued till BC_3F_5 (Fig. 1) where pure homozygous lines for all three resistance genes were detected. The selected and confirmed pyramided restorer lines were used for pathogenicity test against five *BXo* races.

Fig. 1. Schematic depiction of the introgression of *xa5, xa13* and *Xa21* into the restorer line

DNA extraction and PCR amplification

Leaves of twenty-one days old seedlings were obtained from the field to extract DNA. The adapted CTAB (Cetyl Trimethyl Ammonium Bromide) protocol described by Nihad et al., [38] was used for DNA extraction. DNA quality was measured by a nanodrop spectrophotometer (Nanodrop^{TM} 2000/2000c) and kept at -20°C for further use. Four linked/gene-specific molecular markers, MP1-F & MP2-R, *xa5*_F2_Sus. Forward + *xa5*_F2_res. Forward + *xa5*_ R2 reverse primer (common), xa13F_130_147 + xa13R_1678_1662 and pTA248 linked to *Xa4, xa5, xa13* and *Xa21*, respectively, were used for foreground selection in each backcross and selfing population**.** PCR components, 3 µL DNA, 1 µL primer (0.5µL forward and 0.5µL reverse primers), 5 µL GoTaq® G2 Green Master Mix (2X) (Promega, USA) and 1.0µL double distilled water (nuclease-free) water were taken in 0.2mL PCR tube to conduct the PCR reaction. Details of gene-linked/specific primers and their PCR program are given in **(**Table 1 and Supplementary Table 1**)**. To visualize the DNA band, electrophoresis was done on 1.5% agarose gel followed by ethidium bromide staining.

Table 1. Molecular markers used for foreground selection.

Gene	Chr	Marker	Sequences (5'-3')	Expected allele size (bp)	Reference
Xa4	11	MP1	F-ATCGATCGATCTTCACGAGG	150	Suh et al., 2013
		MP2	R-TGCTATAAAAGGCATTCGGG		
xa5	5	*xa5*_F2	SF-GCTCGCCATTCAAGTTCTTGTC	198	IRRI, 2013
		*xa5*_F2	RF-GCTCGCCATTCAAGTTCTTGAG		
		*xa5*_R2 (Common)	RP-CCTTGATAGAAACCTTGCTCTTGAC		
xa13	8	*xa13F*_130_147	F-CCTGATATGTGAGGTAGT	1520	Kumar et al., 2013

		*xa13R*_1678_1662	R-GAGAAAGGCTTAAGTGC		
Xa21	11	pTA248	F-AGACGCGGAAGGGTGGTTCCCGGA	1000	Xu et al., 2013
			R-AGACGCGGTAATCGAAAGATGAAA		
Rf3	1	RM1	F-GCGAAAACACAATGCAAAAA	115	Revathi et al., 2013
			R-GCGTTGGTTGGACCTGAC		
Rf4	10	DRCG-RF4-14F &14R	F-GCAATGCTTGTATTCAGCAAA	800	Suresh et al., 2012
			R-TCCAGCTGTAAATCCGTCAA		

Validation of pyramided restorer lines by SNP markers

Finally, selected pyramided restorer lines were further validated by SNP markers against 20 QTL traits which are shown the Supplementary Table 2.

Molecular Study for fertility status of pyramided restorer lines

Selected five pyramided restorer lines with one fertility restorer and non-fertility restorer lines were used as a check to confirm fertility restorer genes, *Rf3* and *Rf4* with restorer allele through molecular markers (Table 1) during Transplanted Aman season, 2020 (wet season).

Conventional approaches to study fertility status of pyramided restorer lines

Constitute of source nursery

A total of five pyramided restorer lines along with two wild abortive (WA) CMS lines, IR75608A and IR79156A were constituted in the source nursery during the Boro season (dry season), 2020. Each line was grown in three (3) sets with different planting times for proper synchronization of flowering to make a test cross for the validation of fertility status.

Evaluation of testcross F_1s

A total ten (10) test cross F_1s (**Supplementary Table 3**) were evaluated in test cross nursery by two ways such as a microscopic study and phenotypic study for confirmation of fertility status at the flowering stage by following the Hybrid Rice Breeding Manual, IRRI, Philippines 1997 i.e., Full fertile (FF): 81-100% fertility, Fertile:61-80%, Partially fertile (PF):31-60%, Partially sterile (PS):21-20%, Sterile (S): 1-20% and Completely sterile (CS): 0% fertility.

Pollen fertility and spikelet fertility status of testcross f_1s

The pollen grains were put on a glass slide and dyed with one drop of 1% Iodine Potassium Iodide (IKI) solution. The anthers were gently smashed with a needle to release pollen grains, and pollen fertility was determined using a compound microscope at 10X

magnification. Spikelet fertility percentage (seed sett %) was counted manually. When the dark black cell was seen in I-KI solution under a microscope, it meant that male parents were confirmed to be suspected restorers. The fertility of pollen and spikelet was measured by the following formula: Pollen fertility (%) = (No. of fertile pollen/No. of total pollen)*100; Spikelet fertility (%) = (Filled spikelets per panicle/Total spikeletsper panicle)*100.

Screening for BB Resistance

The parent and selected pyramided restorer lines were inoculated with five bacterial blight races (*BXo93, BXo220, BXo826, BXo822 and BXo887*) suspension following the adapted clipping procedure of Rashid et al., [23] modified from Kauffman et al., [39]. At the maximum tillering stage, ten hills per entry were selected for *BXo* race inoculation and 8-10 leaves were trimmed from the apex. Before plunging into the fresh isolate mixture, the scissors were rinsed three times with sterilized water and then sterilized in 70% ethanol for 30 minutes. Following 14 days of inoculation, eight (8) infected leaves per hill were collected from the field for lesion length measurement. The disease response was graded using the modified scale of Akter et al., [25]. Briefly, disease data was categorized into six scales (0 to 9) i.e., 0-Highly resistant (HR, lesion length <0.5 cm), 1-Resistant (R, lesion length 0.5-3.0 cm), 3-Moderately resistant (MR, lesion length 3.1-5.0 cm), 5-Moderately susceptible (MS, lesion length 5.1-10.0 cm), 7-Susceptible (S, lesion length 10.1-15.0 cm) and 9-Highly susceptible (HS lesion length >15.0 cm).

Agro-morphological traits and grain quality test of pyramided restorer lines

Five pyramided restorer lines and their recurrent parent were seeded in the Transplanted Aman season (wet season) of 2020. Twenty-one days old seedling was transplanted with a spacing of 20 cm × 15 cm and other agronomic practices. Data was noted on five plants from each pyramided restorer line in BC_3F_5 generation for agronomic traits like plant height (cm), days to 50% flowering (day), days to maturity (day), number of effective tiller/hill, panicle length (cm), number of spikelet/panicle, spikelet fertility (%), 1000 grain weight (g), Grain yield/ hills (g). On the other hand, grain quality parameters such as milling outturn, head rice recovery, milled rice length, milled rice breadth, length breadth ratio, amylose content, protein content, cooking time, elongation ratio and imbibition ratio were measured in the laboratory of grain quality and nutrition department of Bangladesh Rice Research Institute, Gazipur, during 2020.

Statistical analysis of the data

Replication means data of the yield contributing traits and physicochemical and cooking characters under the study were subjected to univariate analysis. Univariate analysis of the individual character was done by computer using Excel program 2010 software. Statistical analysis was performed with independent samples using least of significance difference (LSD) at a 5% level and coefficient of variation percentage (CV%). Selected five pyramid restorer lines were genotyped with 20 trait-based single nucleotide polymorphism (SNP) markers developed by the International Rice Research Institute (IRRI; https://www.irri.org/; https://gsl.irri.org/) [40] utilizing Kompetitive allele-specific PCR (KASP) assay for high-precision bi-allelic characterization of SNP with Intertek (https://www.intertek.com/agriculture/agritech/) as a service provider. The SNP markers associated with the trait of interests such as snpOS00493, snpOS00061, snpOS00054 for BB resistant; snpOS00478, snpOS00451, snpOS00006, snpOS00468 for blast resistant; snpOS00430 and snpOS00442 for brown planthopper; snpOS00466, snpOS00467 for gall midge; snpOS00445, snpOS00446, snpOS00038 for amylose content; snpOS00024 for chalkiness (grain quality); snpOS00396 for grain number and snpOS00409, snpOS00410, snpOS00411, snpOS00397 for salt were assayed.

Results

Validation of markers for foreground selection of parents

The repeatability and polymorphism of gene-based/linked markers were checked between the recurrent parent, BRRI31R and donor parent IRBB60 (Table 2). Among the parents, the restorer line BRRI31R showed monomorphic nature with a resistant allele (150bp) of the *Xa4* gene but showed susceptible alleles (198bp, 1322bp and 650bp) for *xa5*, *xa13* and *Xa21* genes with polymorphic nature respectively. Gel photographs of the markers for the four bacterial blight resistance genes in the parents are presented in Fig. 2a.

Fig. 2. (a) Gel photographs showing banding pattern of the markers for the four bacterial blight (BB) resistance genes such as *Xa4*, *xa5*, *xa13* and *Xa21* in the parents. **(b)** Phenotypic

screening of selected parents along with resistant and susceptible check against *BXo* races of bacterial blight (BB) disease.

Table 2. Evaluation of parents for BB resistance genes *Xa4, xa5, xa13* and *Xa21* genes using MAS and artificial inoculation

**Sl.**	**Parents**	**BB resistance genes**				**Isolates/Lesion length (cm)**					**Mean Lesion length (cm)**	**Disease reaction**
		Xa4	***xa5***	***xa13***	***Xa21***	***BXo 93***	***BXo 220***	***BXo 822***	***BXo 826***	***BXo 887***		
1	BRRI31R (RP)	P	A	A	A	21.7	24.1	23.2	25.1	23.6	23.5	HS
2	IRBB60 (DP & RC)	P	P	P	P	1.0	0.8	0.8	0.9	0.8	0.9	R
3	IR24(SC)	A	A	A	A	20.9	22.7	25.1	25.0	25.6	23.8	HS

Pathogenicity test of parents against bacterial blight pathogen

After the morphological screening, the recipient parent, BRRI31R showed (23.5 cm) similar mean lesion length compared to susceptible check IR24 (23.8 cm) while the resistant check showed mean lesion length 0.9 cm (Table 2). The disease reaction of parents along with susceptible check against bacterial blight pathogen is shown in Fig. 2b**.**

Fig. 2. (a) Gel photographs showing banding pattern of the markers for the four bacterial blight (BB) resistance genes such as *Xa4*, *xa5*, *xa13* and *Xa21* in the parents. **(b)** Phenotypic screening of selected parents along with resistant and susceptible check against *BXo* races of bacterial blight (BB) disease.

Stacking of bacterial blight resistance genes into BRRI31R using MABC

Fifty heterozygous F_1 plants obtained from the cross between BRRI31R × IRBB60 were confirmed through molecular markers. Out of fifty, ten heterozygous plants with three BB resistance genes (*xa5*, *xa13* and *Xa21*) were backcrossed to respective recurrent parent (BRRI31R). In BC_1F_1, foreground selection with target genes resulted in the selection of heterozygous plants. A total of 24 out of 55 heterozygous plants were found with triple BB resistance genes and these plants were backcrossed with a recurrent parent to obtain BC_2F_1 populations. In BC_2F_1, a total of 22 out of 62 plants were found with three resistant alleles in heterozygous condition for *xa5, xa13* and *Xa21* genes and these plants were further backcrossed to produce BC_3F_1 populations. From BC_3F_1 populations, 24 out of 65 plants were selected with

three BB resistance genes and selfed to produce BC_3F_2 progenies. Finally, two hundred BC_3F_2 progenies were tested using linked markers to identify homozygous plants for different R genes of BRRI31R × IRBB60. A gel photograph of the BC3F2 population is presented in. Plants with single, double and triple gene combinations with BB resistance were identified in this study. Among them, 45 plants were shown with homozygous resistance for *xa5*, 52 for *xa13* and 49 for *Xa21* respectively. Simultaneously, 23 plants for *xa5* +*xa13*, 25 plants for *xa5* + *Xa21*, 17 for *xa13*+*Xa21* and 15 for *xa5* + *xa13* + *Xa21* genes were found homozygous alleles. One hundred and seventy plants were found homozygous to susceptible alleles like as recurrent parent. Finally, five (5) homozygous plants for three resistance genes (*xa5*, *xa13* and *Xa21*) were selected based on molecular screening and phenotypically compared to respective recurrent parents. The segregation of bacterial blight resistance in BC_3F_2 populations was analyzed using the chi-square ($\chi 2$) test. The χ^2 values value of segregation analysis for *xa5*, *xa13* & *Xa21* genes was insignificant as the calculated χ^2 values, 2.920 (*xa5*), 1.720 (*xa13*) and 2.110 (*Xa21*) is lesser than the tabulated values at 5% (5.991) and 1% (9.210) levels (Table 3). The selected five homozygous BC_3F_2 plants were forwarded to BC_3F_3 and continued from BC_3F_3 to BC_3F_5 progenies. BRRI31R had by default *Xa4* gene (Fig. 2a**)** and for this reason, further confirm the four BB resistance genes (*Xa4*, *xa5*, *xa13* and *Xa21)* through gene-based/linked markers in the cross combination, BRRI31R × IRBB60. Five pyramided restorer lines with four (4) BB resistance genes were confirmed through molecular markers (Figs. 3a-d) and these lines were used in the evaluation nursery for pathological screening against five *BXo* races and the confirmation of fertility status. Validation of BB resistance genes with additional traits using 20 SNP panels by molecular approach and also agro-morphological traits with grain quality test were evaluated.

Fig. 3. Gel image of *Xa4* (a), *xa5* (b), *xa13* (c), *Xa21* (d), *Rf3* (e) and *Rf4* (f) genes in fixed restorer lines of BC_3F_5. Here, L1, 100bp; L2, 1kb; L3, 50bp; P1, BRRI31R (Recipient Parent); P2, IRBB60 (Donor Parent); P3, IRBB4 (resistant check for *Xa4* gene); P4, IR24 (susceptible check); P5, IRBB5 (resistant check for *xa5* gene); P6, IRBB13 (resistant check for *xa13* gene); P7, IRBB21 (resistant check for *Xa21* gene); P8, IR96479-81-7-1-1-B-1-1-1-R (RA, restorer allele); P9, IR58025A (NR, non-restorer allele).

Table 3. Marker analysis in segregating BC_3F_2 populations for *xa5*, *xa13* and *Xa21* gene

BRRI31R x IRBB60						
**Genes/Makers**	**Observed value**		**Expected ratio**	**Expected value**	**χ^2 - value**	**Probability**
xa5	RR= R	45	1	50	0.500	0.232
	Rr= H	112	2	100	1.440	

	rr = S	43	1	50	0.980	
**Total**		200	4	200	**2.920**	
xa13	RR= R	52	1	50	0.080	0.423
	Rr= H	106	2	100	0.360	
	rr = S	42	1	50	1.280	
Total		200	4	200	1.720	
Xa21	RR= R	49	1	50	0.020	0.348
	Rr= H	109	2	100	0.810	
	rr = S	42	1	50	1.280	
**Total**		200	4	200	2.110	

Here, R: resistant; S: susceptible; H: segregant; RR: plants with a banding pattern a like to the resistant parent alleles; Rr: heterozygous plants; rr: plants with a banding pattern a like to the susceptible parent alleles; χ^2 : actual value of the chi-square test for resistant/susceptible ratio.

Selected pyramid lines screening against bacterial blight pathogen

The BB resistance of pyramided restorers with the parents and susceptible check IR24 were evaluated by five *BXo* races. The mean lesion length of pyramided restorers (*Xa4, xa5, xa13* and *Xa21)* was very less (<3 cm) compared to recurrent parents and susceptible check IR24 (Fig. 4). Out of five, two pyramid restorer lines, BRRI31R-MASP3 and BRRI31R-MASP4 were showed highly resistant reaction against all the races (*BXo93, BXo220, BXo826, BXo822 and BXo887)* with mean lesion length <0.5 cm which was similar to the mean lesion of the donor parent IRBB60. This result indicates that the introgression of *xa5, xa13* and *Xa21* genes significantly enhanced the resistance of the improved pyramided restorer lines to BB. The mean lesion length (cm) of all pyramided restorers lines (resistant to highly resistant) with parents and susceptible checks are shown in Table 4**.**

Fig. 4. Phenotypic screening of selected pyramiding restorer lines along with respective recurrent parent, donor parent and susceptible check against *BXo* races of bacterial blight (BB) disease.

Table 4. Evaluation of pyramided restorer lines and parents against bacterial blight pathogen

**Pyramided restorer lines with parents and sus.ck**	**Lesion length (cm)**					**Mean lesion length (cm)**	**Disease reactio n**
	BXo93	***BXo220***	***BXo822***	***BXo826***	***BXo887***		
BRRI31R-MASP1	1.0	1.1	1.5	1.2	1.6	1.3	R
BRRI31R-MASP2	1.2	1.5	1.4	1.1	1.0	1.2	R
BRRI31R-MASP3	0.2	0.3	0.4	0.1	0.5	0.3	HR
BRRI31R-MASP4	0.1	0.2	0.3	0.4	0.2	0.2	HR
BRRI31R-MASP5	1.1	0.9	1.4	1.3	1.5	1.2	R
IRBB60-DP	0.2	0.3	0.4	0.1	0.5	0.3	HR
BRRI31R-RP	22.7	24.5	23.8	25.5	24.2	24.1	HS
IR24 - Sus.ck	24.7	25.6	26.1	25.2	26.5	25.6	HS

Here, DP-Donor parent; RP-Recurrent parent; SC-Susceptible check; R-Resistant; HR- Highly rsistant; HS- Highly susceptible.

Evaluation of fertility status of pyramided restorer lines

Fertility status observed by gene-based/linked markers

Five pyramid restorer lines were subjected to PCR analysis using gene-based or linked markers against fertility restore genes *Rf3* & *Rf4*. All pyramid restorer lines showed restorer alleles, *Rf3* and *Rf4* where band size was 115bp and 800bp, respectively (Figs. 3e-f). So, these lines had the ability to restore fertility.

Fig. 3. Gel image of *Xa4* (a), *xa5* (b), *xa13* (c), *Xa21* (d), *Rf3* (e) and *Rf4* (f) genes in fixed restorer lines of BC_3F_5. Here, L1, 100bp; L2, 1kb; L3, 50bp; P1, BRRI31R (Recipient Parent); P2, IRBB60 (Donor Parent); P3, IRBB4 (resistant check for *Xa4* gene); P4, IR24 (susceptible check); P5, IRBB5 (resistant check for *xa5* gene); P6, IRBB13 (resistant check for *xa13* gene); P7, IRBB21 (resistant check for *Xa21* gene); P8, IR96479-81-7-1-1-B-1-1-1-R (RA, restorer allele); P9, IR58025A (NR, non-restorer allele).

Fertility status was observed of testcrosses F_1s by the conventional way

Out of ten, four test crosses of F_1s combinations, IR75608A × BRRI31R-MASP3, IR75608A × BRRI31R-MASP4, IR79156A × BRRI31R-MASP3 and IR79156A × BRRI31R-MASP4 were found having the highest pollen fertility (90.45%, 91.75%, 94.15% and 92.89%, respectively) and seed sett (94.64%, 93.88%, 95.10% and 94.46%, respectively) percentage (Table 5). F_1 seed sett or spikelet fertility of these test cross above 90% indicating that these were pyramided restorer lines (BRRI31R-MASP3 and BRRI31R-MASP4) denoted strong fertility status with restorer genes *Rf3* and *Rf4* against IR75608A and IR79156A.

Table 5. Restoration confirmation of pyramided restorer lines

Sl.	Testcross F_1s of pyramided lines	Pollen Fertility (%)	Spikelet fertility or Seed Set %)	Category	Remarks
1	IR75608Ax BRRI31R-MASP1	87.20	89.80	FF	Restorer
2	IR75608Ax BRRI31R-MASP2	86.00	87.20	FF	Restorer
3	IR75608Ax BRRI31R-MASP3	90.45	94.64	FF	Restorer
4	IR75608Bx BRRI31R-MASP4	91.75	93.88	FF	Restorer
5	IR75608B x BRRI31R-MASP5	88.22	88.35	FF	Restorer
6	IR79156Ax BRRI31R-MASP1	89.22	91.78	FF	Restorer
7	IR79156Ax BRRI31R-MASP2	91.95	92.50	FF	Restorer
8	IR79156Ax BRRI31R-MASP3	94.15	95.10	FF	Restorer
9	IR79156Ax BRRI31R-MASP4	92.89	94.46	FF	Restorer
10	IR79156Ax BRRI31R-MASP5	93.33	91.99	FF	Restorer

Here, FF: Full Fertile

Pyramided restorer lines validated by SNP markers

Selected five pyramid restorer lines were validated by SNP markers linked with 20 QTL of rice traits. Two pyramided restorer lines, BRRI31R-MASP3 and BRRI31R-MASP4 consisted of target QTL *xa5* (resistant allele AG:AG by snpOS00054), *xa13* (resistant allele C:C by snpOS000493), *Xa21* (resistant allele C:C by snpOS00061), *Pita* (resistant allele C:C by snpOS00006), *Chalk5* (resistant allele G: G by snpOS00024) but another three pyramid restorer lines, BRRI31R-MASP1, BRRI31R-MASP2 and BRRI31R-MASP5 contained all of the above genes without blast resistance gene *Pita* (resistant allele C:C by snpOS00006) (Table 6). After pathogenicity test and validation with molecular and morphological study, out of five, two pyramided restorer lines, BRRI31R-MASP3 and BRRI31R-MASP4 were selected based on the best phenotypic similarity to the recurrent parent with blast resistance gene, *Pita*. For the sake of clarification, disease reaction of the *Pita* gene was not tested here.

Table 6. List of pyramided restorer lines along with parents and an important trait

SL	Pyramided restorers	Important traits present
1	BRRI31R-MASP1	*Xa4*, *xa5*, *xa13*, *Xa21*, Chalk5, *Rf3*, *Rf4*
2	BRRI31R-MASP2	*Xa4*, *xa5*, *xa13*, *Xa21*, Chalk5, *Rf3*, *Rf4*
3	BRRI31R-MASP3	*Xa4*, *xa5*, *xa13*, *Xa21*, Pita, Chalk5, *Rf3*, *Rf4*
4	BRRI31R-MASP4	*Xa4*, *xa5*, *xa13*, *Xa21*, Pita, Chalk5, *Rf3*, *Rf4*
5	BRRI31R-MASP5	*Xa4*, *xa5*, *xa13*, *Xa21*, Chalk5, *Rf3*, *Rf4*
6	BRRI31R-RP	*Xa4*, Chalk5, Pita, *Rf3*, *Rf4*
7	IRBB60-DP	*Xa4*, *xa5*, *xa13*, *Xa21*, Chalk5

Here, RP: Recurrent Parent, DP: Donor Parent

Agro-morphological and grain quality test of pyramided restorer lines

No significant differences were found between the pyramided restorer lines and the respective recurrent parent. All the selected pyramided restorer lines had more or alike agro-morphological and grain-quality characters as recipient parent (Table 7, Table 8 and Fig. 5). Out of the five two pyramided restorer lines, BRRI31R-MASP3 and BRRI31R-MASP4 produced higher yields than the recipient parent.

Fig. 5. Phenotype of the selected five pyramided restorer lines in BC_3F_5 progenies compared with recurrent parent BRRI31R.

Table 7. Agro-morphological traits of pyramided restorer lines and recurrent parent

**Pyramided lines and recurrent parent**	**PH (cm)**	**DFF**	**DTM**	**ET/P**	**PL (cm)**	**G/P**	**SF (%)**	**TGW (g)**	**GY/P (g)**
BRRI31R-MASP1	115.2	107	132	9	28.3	215	91	31.8	38.8
BRRI31R-MASP2	114.9	108	133	10	28.4	212	93	31.5	39.5
**BRRI31R-MASP3**	115.0	105	130	12	28.8	225	95	32.0	42.2
**BRRI31R-MASP4**	115.3	102	127	11	28.7	220	94	31.9	41.5
BRRI31R-MASP5	115.5	106	131	10	28.5	218	90	31.7	40
BRRI31R-RP	115.1	109	134	10	28.5	208	93	32.2	40.2
Mean	115.2	106.2	131.2	10.3	28.5	216.3	92.7	31.9	40.4
LSD (5%)	0.21	2.39	2.39	0.99	0.18	5.79	1.79	0.23	1.22
CV(%)	0.17	2.14	1.73	9.12	0.60	2.54	1.83	0.70	2.86

Here, PH: Plant height; DFF: Days to 50% Flowering; DTM: Days to maturity; ET/P: Effective tiller/Plant; PL: Panicle length in cm; G/P: Grains per panicle; SF: Spikelet fertility in percent; TGW: Thousand grain weight (g); GY/P: Grain yield per plant (g); LSD: Least of Significant Difference at 5% probability level; CV: Coefficient of Variation.

Table 8. Mean performance of grain quality parameters of pyramided restorer lines and parent

Pyramided lines	MO (%)	HRR (%)	ML (mm)	MB (mm)	L/B ratio	AC (%)	PC (%)	CT (min.)	ER	IR
BRRI31R-MASP1	73.90	72.00	7.00	2.20	3.18	22.90	7.90	17.20	1.30	4.45
BRRI31R-MASP2	74.00	71.70	7.10	2.20	3.23	23.05	8.00	17.88	1.31	4.48
BRRI31R-MASP3	74.80	73.00	7.30	2.29	3.19	23.38	8.20	18.10	1.34	4.50
BRRI31R-MASP4	74.50	73.00	7.22	2.30	3.14	23.30	8.08	18.25	1.32	4.51
BRRI31R-MASP5	73.70	71.50	7.00	2.20	3.18	23.08	7.80	17.84	1.29	4.45
BRRI31R -RP	74.00	72.00	7.20	2.30	3.13	23.00	7.90	18.30	1.30	4.50
Mean	74.15	72.20	7.14	2.25	3.18	23.12	7.98	17.93	1.31	4.48
LSD (5%)	0.44	0.68	0.13	0.06	0.04	0.19	0.15	0.42	0.02	0.03
CV(%)	0.56	0.90	1.73	2.36	1.14	0.80	1.81	2.25	1.37	0.59

MO: Milling outturn in percent; HRR: Head rice recovery; ML: Milled rice length in mm; MB: Milled rice breadth in mm; L/B: Length breadth ratio; AC: Amylose content in percent; PC: Protein content in percent; CT: Cooking time; ER: Elongation ratio and IR: Imbibition ration.

Discussion

BB disease is a key limitation for hybrid rice farming in Bangladesh since most hybrid rice parental lines are sensitive to BB. Introgression of bacterial blight resistance genes into parental lines of hybrid rice using MABC is the best way to develop durable bacterial blight resistance lines. Because through MABC, the desired genes can be easily transferred into the

elite lines and can quickly recover the recurrent parent characteristics with a minimum number of backcrosses. We developed five pyramided lines having *Xa4*, *xa5*, *xa13* and *Xa21* genes with fertility restorer genes. Many reviewers previously studied and introgressed BB resistance genes into promising to restore lines for the development of BB resistant pyramid hybrid rice [41–44]. MAS offers a simpler and more efficient method to improve rice cultivars or lines and marker-assisted introgression of major genes/QTLs has helped to develop resistant varieties against major diseases such as BB, blast, tungro etc. [8,30,45–47]. But conventional backcross cannot precisely transfer more than one gene into the cultivars and also the process needs a significant amount of time [48–50]. In this study, selected parents, BRRI31R and IRBB60 were validated against four genes–based or genes-linked markers for foreground selection. It was observed that the restorer line, BRRI31R showed monomorphic nature with a resistant allele against the *Xa4* gene but showed a susceptible allele against the *xa5, xa13* and *Xa21* genes.

In this study, BC_3F_5 pyramid lines have been developed by backcrossing and selfing, and at each stage, BB resistant plants were selected based on molecular screening by MAS, pathogenicity test and phenotype comparison to their respective recurrent parent. The similar approach followed by earlier researchers and successfully introgressed two or three or four BB resistance genes into the rice cultivars or lines [29,51–53]. Our restorer lines were selected with target BB resistance genes using molecular markers and so there is a chance to carry linkage drag with the target gene. Backcross breeding is helpful to remove the linkage drug [54,55] and to eliminate linkage drag, backcross progenies were selected in each backcross and segregating generation through foreground and phenotypic selection. Therefore, it was proceeded by three backcrosses (BC_3) and advanced BC_3F_1 to BC_3F_5 generation and foreground selection was performed up to BC_3F_5 generations to ensure the presence of the desired genes and phenotypically compared to respective recurrent parents.

In the present study, the segregated populations were analyzed in BC_3F_2 progenies against the *xa5, xa13* and *Xa21* genes by chi-square ($\chi 2$) test. Based on gene combinations of cross combination BRRI31R × IRBB60, it was observed that the segregated genotypic ratio of 1:2:1 which fitted expected segregation ratio for single gene model. The inheritance pattern of BB resistance controlled by single gene and segregation analysis on *xa5, xa13* and *Xa21* genes revealed that the chi-square ($\chi 2$) value is lower than the tabulated value at 5% (5.99) and 1% (9.21) levels which prove that these genes individually followed the first law of Gregor Johan Mendel [56–58].

Five homozygous pyramided restorer lines were selected from the cross combination, BRRI31R × IRBB60, with three BB resistance genes, *xa5 + xa13 + Xa21* in BC_3F_5 progenies which showed highly resistant reaction against BB pathogens. Combination of more than one resistance gene is effective to defend against BB pathogen and gene combination of *xa5 +xa13 + Xa21* are effective against the wide range of BB isolates of India [53,59–61]. It was noted that BRRI31R had the *Xa4* gene by default and for this reason, finally, five homozygous pyramided restore lines were selected with four BB resistance genes, *Xa4 + xa5 + xa13 + Xa21* from the cross combination, BRRI31R × IRBB60 in BC_3F_5 progenies. Though *Xa4* is not effective against the BB pathogen of Bangladesh but mbination of this gene might enhance the function of other resistance gene and a combination of these four genes are also very effective against the diverse isolates of BB pathogens [23,25,34,59,62]. These gene combinations also gives long-lasting and broad-spectrum resistance in the R8012 restorer line for Chinese CMS Zhong-9A restoration [63].

All selected pyramided restorer lines with respected recurrent parent were screened against five *BXo* races. It was observed that all pyramided lines showed resistance to highly resistance to all five races of *Xoo* and mean lesion length below 3 cm and ranged from 0.2 to 1.3 cm. More than one gene, specifically a combination of *xa5+xa13+Xa21* is effective against more than one BB virulent isolates of Bangladesh [25,34]. The data indicates that pyramided BB resistance genes in parental lines of rice hybrid could improve resistance to *Xoo*. Four gene combinations *(Xa4 + xa5 + xa13 + Xa21*) showed highly resistant and comparatively showed a higher degree of resistance than parent and susceptible check and findings are consistent with previous investigations [23,30,33,53,59].

The discovery of restorers is critical for the commercialization of heterosis breeding programs in hybrid rice utilizing the cytoplasmic male sterility (CMS) method [64]. In this study, all five pyramided restorer lines viz., BRRI31R-MASP1, BRRI31R-MASP2, BRRI31R-MASP3, BRRI31R-MASP4 and BRRI31R-MASP5 showed fertility restorer alleles of *Rf3* and *Rf4*. F_1s pollens were found and of these F_1s pollen were studied under compound microscopic and seed set (%) was observed under field conditions. Under both observations, all restorer lines displayed full fertility. So, all pyramided restorer lines possessed fertility restoring ability and it proves that the resistance genes of the study had not any negative effect on the restoring ability [44,65]. These pyramided restorer lines will be used in line × tester analysis for finding the suitable combiner with the best heterotic combination compared to standard checks by General combining ability (GCA) and specific combining ability (SCA) analysis. After molecular

screening, selected 5 pyramided restorer lines were screened against 20 QTLs trait using SNP markers. In this study, out of 20, 5 QTLs were also confirmed.

The agronomic characters analysis revealed that BC_3F_5 restorer lines derived from the BRRI31R were more or less similar to the recurrent parent and advanced lines of the backcross population are similar to the recipient parent because of background recovery [33]. However, out of five, two pyramided restorer lines produced higher grain yields per plant than the recurrent parent. In addition, all pyramided restorer lines were not significantly different in milling outturn in percent, head rice recovery, milled rice length, milled rice breadth, length breadth ratio, amylose content, protein content, cooking time, elongation ratio and imbibition ratio, indicating that BC_3F_5 pyramiding lines had grain quality consistent with BRRI31R. The findings revealed that there were no decreases in yield or grain quality as a result of the pyramiding of the four BB resistance genes (*Xa4, xa5, xa13 and Xa21*).

Conclusion

We have successfully introgressed BB resistance genes into a promising restorer line, BRRI31R using MABC. The pyramided restorer lines, BRRI31R-MASP1, BRRI31R-MASP2 and BRRI31R-MASP5 consisted of *Xa4, xa5, xa13, Xa21*, *Chalk5*, *Rf3* and *Rf4* genes. Moreover, BRRI31R-MASP3 and BRRI31R-MASP4 consisted of blast resistance gene *Pita* with *Xa4, xa5, xa13, Xa21, Chalk5, Rf3* and *Rf4* genes and these two pyramided restorer lines showed high resistant reactions against *BXo* races. It also demonstrated the utility of foreground selection in discovering target genes for bacterial blight resistance. These studies, concluded that the developed pyramided restorer lines, BRRI31R-MASP3 and BRRI31R-MASP4 with fertility restore genes would be used as donor parent to transfer bacterial blight resistance genes from pyramided restorer lines into highly susceptible restorer lines of promising hybrid rice. These lines, BRRI31R-MASP3 and BRRI31R-MASP4 might be used for parental lines improvement program by (R x R) crossing based on desired traits and also be used as a male parent for the development of BB resistant heterotic hybrids. The newly developed resistant lines could be also a replacement of our released popular hybrid rice variety of BRRI hybrid dhan5 and BRRI hybrid dhan7.

Acknowledgements

The authors would like to express their gratitude to the Project Director for the financial assistance provided by the Strengthening Physical Infrastructure and Research Activities (SPIRA) Project, Bangladesh Rice Research Institute, through the Ministry of Agriculture,

Bangladesh. The authors are highly grateful to Plant Pathology Division and Hybrid Rice Division for providing all necessary support. The authors are extremely thankful to the Plant Pathology Division and the Hybrid Rice Division for providing all required assistance.

Competing Interest

Authors declare that they have no any competing interest.

Funding

Authors received funds from Strengthening Physical Infrastructure and Research Activities (SPIRA) Project, Bangladesh Rice Research Institute, through the Ministry of Agriculture, Bangladesh to conduct this experiment.

Data availability

The datasets generated during and/or analyzed during the current study are available from the corresponding authors on reasonable request.

References

1. Mamun MA Al, Nihad SAI, Sarkar MAR, Aziz MA, Qayum MA, Ahmed R, et al. Growth and trend analysis of area, production and yield of rice: A scenario of rice security in Bangladesh. Aschonitis VG, editor. PLoS One. 2021;16: e0261128. doi:10.1371/JOURNAL.PONE.0261128

2. Kabir MS, Salam M, Islam A, Sarkar MAR, Mamun M, Rahman M, et al. Doubling Rice Productivity in Bangladesh: A Way to Achieving SDG 2 and Moving Forward. Bangladesh Rice J. 2020;24: 1–47. doi:10.3329/BRJ.V24I2.53447

3. Kabir M, Salam M, Chowdhury A, Rahman N, Iftekharuddaula K, Rahman M, et al. Rice Vision for Bangladesh: 2050 and Beyond. Bangladesh Rice J. 2016. doi:10.3329/brj.v19i2.28160

4. Mezanur-Rahman M, Anamul-Haque M, Arafat-Islam-Nihad S, Mahmudul-Hasan-Akand M, Ruhul-Amin-Howlader M. Morpho-physiological response of Acacia auriculiformis as influenced by seawater induced salinity stress. For Syst. 2016;25: e071. doi:10.5424/fs/2016253-09386

5. Mamun A Al, Rahman MNF, Abdullah Aziz M, Qayum MA, Hossain MI, Nihad SAI, et al. Identification of Meteorological Drought Prone Area in Bangladesh using Standardized Precipitation Index. J Earth Sci Clim Change. 2018;09. doi:10.4172/2157-7617.1000457

6. Rout D, Jena D, Singh V, Kumar M, Arsode P, Singh P, et al. Hybrid Rice Research: Current Status and Prospects. Recent Adv Rice Res. 2020 [cited 1 Oct 2022]. doi:10.5772/INTECHOPEN.93668

7. Qian Q, Zhang F, Xin Y. Yuan Longping and Hybrid Rice Research. Rice. 2021;14: 1–3. doi:10.1186/S12284-021-00542-4/METRICS

8. Nihad SAI, Hasan MK, Kabir A, Hasan MAI, Bhuiyan MR, Yusop MR, et al. Linkage of SSR markers with rice blast resistance and development of partial resistant advanced lines of rice (Oryza sativa) through marker-assisted selection. Physiol Mol Biol Plants 2022 281. 2022;28: 153–169. doi:10.1007/S12298-022-01141-3

9. Latif MA, Nihad SAI, Mian MS, Akter S, Khan MAI, Ali MA. Interaction among sheath diseases complex of rice and ribosomal DNA analysis for the differentiation of

Rhizoctonia solani, R. oryzae and R. oryzae-sativae. Plant Stress. 2022;5: 100100. doi:https://doi.org/10.1016/j.stress.2022.100100

10. Sarker MAR, Alam K, Gow J. Exploring the relationship between climate change and rice yield in Bangladesh: An analysis of time series data. Agric Syst. 2012. doi:10.1016/j.agsy.2012.06.004

11. Nihad S, Ara A, Rashid M, Hasan M, Khan M, Latif M. Genetic Divergence of Rice Genotypes Revealed by Bacterial Blight Disease and Morphological Traits. Bangladesh Rice J. 2021. doi:10.3329/brj.v24i1.53241

12. Nihad SAI, Manidas AC, Hasan K, Hasan MAI, Honey O, Latif MA. Genetic variability, heritability, genetic advance and phylogenetic relationship between rice tungro virus resistant and susceptible genotypes revealed by morphological traits and SSR markers. Curr Plant Biol. 2021;25: 100194. doi:10.1016/J.CPB.2020.100194

13. Nessa B, Salam MU, Haque AHMM, Biswas JK, Latif MA, Ali MA, et al. Rice False Smut Disease at Different Flowering Times. Bangladesh Rice J. 2015;19: 28–35. doi:10.3329/BRJ.V19I2.28162

14. Alam MS, Islam MR, Hossain I, Bhuiyan MR, Khan MAI. Pathotypic variation of Xanthomonas oryzae pv. oryzae in Bangladesh. Arch Phytopathol Plant Prot. 2016;49: 31–42. doi:10.1080/03235408.2016.1150633

15. M Mubassir MH, Nasiruddin KM, Hoque Shahin N, Nahar Begum S, Kanti Saha M, M Bazlur Rashid AQ, et al. Morpho-Molecular Screening for Bacterial Leaf Blight Resistance in Some Rice Lines and Varieties. http://www.sciencepublishinggroup.com. 2016;4: 146. doi:10.11648/J.JPS.20160406.13

16. Chen LQ, Qu XQ, Hou BH, Sosso D, Osorio S, Fernie AR, et al. Sucrose efflux mediated by SWEET proteins as a key step for phloem transport. Science. 2012;335: 207–211. doi:10.1126/SCIENCE.1213351

17. Verdier V, Vera Cruz C, Leach JE. Controlling rice bacterial blight in Africa: Needs and prospects. J Biotechnol. 2012;159: 320–328. doi:10.1016/J.JBIOTEC.2011.09.020

18. Xia C, Chen H, Zhu X. Identification, Mapping, Isolation of the Genes Resisting to Bacterial Blight and Application in Rice. Mol Plant Breed. 2012. doi:10.5376/mpb.2012.03.0012

19. Ou SH. Exploring Tropical Rice Diseases: A Reminiscence. Annu Rev Phytopathol. 1984;22: 1–11. doi:10.1146/annurev.py.22.090184.000245

20. Mew TW. Focus on Bacterial Blight of Rice. Plant Dis. 1993. doi:10.1094/pd-77-0005

21. Ansari TH, Ahmed M, Akter S, Mian MS, Latif MA, Tomita M. Estimation of Rice Yield Loss Using a Simple Linear Regression Model for Bacterial Blight Disease. Bangladesh Rice J. 2020. doi:10.3329/brj.v23i1.46083

22. Ansari TH, Ahmed M, Akter S, Mian MS, Latif MA, Tomita M. Estimation of Rice Yield Loss Using a Simple Linear Regression Model for Bacterial Blight Disease. Bangladesh Rice J. 2020;23: 73–79. doi:10.3329/brj.v23i1.46083

23. Rashid MM, Nihad SAI, Khan MAI, Haque A, Ara A, Ferdous T, et al. Pathotype profiling, distribution and virulence analysis of Xanthomonas oryzae pv. oryzae causing bacterial blight disease of rice in Bangladesh. J Phytopathol. 2021;169: 438–446. doi:10.1111/jph.13000

24. Ullah I, Jamil S, Iqbal MZ, Shaheen HL, Hasni SM, Jabeen S, et al. Detection of bacterial blight resistance genes in basmati rice landraces. Genet Mol Res. 2012;11: 1960–1966. doi:10.4238/2012.JULY.20.1

25. Akter A, Nihad SAI, Hasan MJ, Hassan L, Robin AHK, Quddus MR, et al. Evaluation of hybrid rice parental lines against bacterial blight disease and detection of resistant gene (s) by gene-specific, linked markers. J Phytopathol. 2022;170: 382–390. doi:10.1111/JPH.13087

26. Rajpurohit D, Kumar R, Kumar M, Paul P, Awasthi A, Osman Basha P, et al. Pyramiding of two bacterial blight resistance and a semidwarfing gene in Type 3 Basmati using marker-assisted selection. Euphytica. 2011;178: 111–126. doi:10.1007/S10681-010-0279-8/TABLES/6

27. Fiyaz RA, Shivani D, Chaithanya K, Mounika K, Chiranjeevi M, Laha GS, et al. Genetic Improvement of Rice for Bacterial Blight Resistance: Present Status and Future Prospects. Rice Sci. 2022;29: 118–132. doi:10.1016/j.rsci.2021.08.002

28. Fiyaz RA, Shivani D, Chaithanya K, Mounika K, Chiranjeevi M, Laha GS, et al. Genetic Improvement of Rice for Bacterial Blight Resistance: Present Status and Future Prospects. Rice Sci. 2022;29: 118–132. doi:10.1016/J.RSCI.2021.08.002

29. Dokku P, Das KM, Rao GJN. Pyramiding of four resistance genes of bacterial blight in Tapaswini, an elite rice cultivar, through marker-assisted selection. Euphytica. 2013. doi:10.1007/s10681-013-0878-2

30. Ramalingam J, Raveendra C, Savitha P, Vidya V, Chaithra TL, Velprabakaran S, et al. Gene Pyramiding for Achieving Enhanced Resistance to Bacterial Blight, Blast, and Sheath Blight Diseases in Rice. Front Plant Sci. 2020. doi:10.3389/fpls.2020.591457

31. Das G, Rao GJN. Molecular marker assisted gene stacking for biotic and abiotic stress resistance genes in an elite rice cultivar. Front Plant Sci. 2015. doi:10.3389/fpls.2015.00698

32. Chen S, Wang C, Yang J, Chen B, Wang W, Su J, et al. Identification of the novel bacterial blight resistance gene Xa46(t) by mapping and expression analysis of the rice mutant H120. Sci Rep. 2020. doi:10.1038/s41598-020-69639-y

33. Hsu YC, Chiu CH, Yap R, Tseng YC, Wu YP. Pyramiding bacterial blight resistance genes in tainung82 for broad-spectrum resistance using marker-assisted selection. Int J Mol Sci. 2020. doi:10.3390/ijms21041281

34. Anik TR, Nihad SAI, Hasan MA-I, Hossain MA, Rashid MM, Khan MAI, et al. Exploring of bacterial blight resistance in landraces and mining of resistant gene(s) using molecular markers and pathogenicity approach. Physiol Mol Biol Plants 2022 282. 2022;28: 455–469. doi:10.1007/S12298-022-01139-X

35. Khan, M. A. I., Kabir, M. S., Monsur M. A., Ali, M. A., & Mia MAT. Pathogenic diversity of Xanthomonas oryzae pv. oryzae in Bangladesh. Bangladesh J Plant Pathol. 2009;25: 1–6.

36. Premkumar A, Kumar M. Applications of molecular markers for bacterial blight resistant varieties in rice (Oryza sativa L.). J Appl Nat Sci. 2017. doi:10.31018/jans.v9i4.1529

37. WANG J, CHENG C, ZHOU Y, YANG Y, MEI Q, LI J, et al. Identification of molecular markers linked to rice bacterial blight resistance genes from Oryza meyeriana. Front Agric Sci Eng. 2015;2: 260. doi:10.15302/J-FASE-2015070

38. Nihad SAI, Kabir A, Rashid M, Honey O, Khan M, Latif M. Comparative assessment of quick and cost-efficient quality DNA extraction methods from rice leaves. J

Bangladesh Agric Univ. 2022;20: 1. doi:10.5455/jbau.107010

39. Kauffman H, Reddy A, Hsieh S, Merca S. An improved technique for evaluating resistance of rice varieties to *Xanthomonas oryzae*. Plant Dis Rep. 1973.

40. Arbelaez JD, Dwiyanti MS, Tandayu E, Llantada K, Jarana A, Ignacio JC, et al. 1k-RiCA (1K-Rice Custom Amplicon) a novel genotyping amplicon-based SNP assay for genetics and breeding applications in rice. Rice. 2019;12: 1–15. doi:10.1186/S12284-019-0311-0/FIGURES/4

41. Sundaram RM, Vishnupriya MR, Biradar SK, Laha GS, Reddy GA, Rani NS, et al. Marker assisted introgression of bacterial blight resistance in Samba Mahsuri, an elite indica rice variety. Euphytica. 2008. doi:10.1007/s10681-007-9564-6

42. Sundaram RM, Vishnupriya MR, Laha GS, Rani NS, Rao PS, Balachandran SM, et al. Introduction of bacterial blight resistance into Triguna, a high yielding, mid-early duration rice variety. Biotechnol J. 2009;4: 400–407. doi:10.1002/BIOT.200800310

43. JI Z juan, Yang S dong, ZENG Y xiang, LIANG Y, YANG C deng, QIAN Q. Pyramiding blast, bacterial blight and brown planthopper resistance genes in rice restorer lines. J Integr Agric. 2016;15: 1432–1440. doi:10.1016/S2095-3119(15)61165-0

44. Balachiranjeevi CH, Bhaskar Naik S, Abhilash Kumar V, Harika G, Mahadev Swamy HK, Sk H, et al. Marker-assisted pyramiding of two major, broad-spectrum bacterial blight resistance genes, Xa21 and Xa33 into an elite maintainer line of rice, DRR17B. PLoS One. 2018;13: e0201271. doi:10.1371/JOURNAL.PONE.0201271

45. Hore TK, Inabangan-Asilo MA, Wulandari R, Latif MA, Nihad AI, Hernandez JE, et al. Introgression of tsv1 improves tungro disease resistance of a rice variety BRRI dhan71. Sci Reports 2022 121. 2022;12: 1–14. doi:10.1038/s41598-022-23413-4

46. Yugander A, Sundaram RM, Singh K, Ladhalakshmi D, Subba Rao L V., Madhav MS, et al. Incorporation of the novel bacterial blight resistance gene Xa38 into the genetic background of elite rice variety Improved Samba Mahsuri. PLoS One. 2018;13: e0198260. doi:10.1371/JOURNAL.PONE.0198260

47. Ramalingam J, Palanisamy S, Alagarasan G, Renganathan VG, Ramanathan A, Saraswathi R. Improvement of stable restorer lines for blast resistance through

functional marker in rice (Oryza sativa L.). Genes (Basel). 2020. doi:10.3390/genes11111266

48. Crossa J, Pérez-Rodríguez P, Cuevas J, Montesinos-López O, Jarquín D, de los Campos G, et al. Genomic Selection in Plant Breeding: Methods, Models, and Perspectives. Trends Plant Sci. 2017;22: 961–975. doi:10.1016/J.TPLANTS.2017.08.011

49. Hasan MM, Rafii MY, Ismail MR, Mahmood M, Alam MA, Abdul Rahim H, et al. Introgression of blast resistance genes into the elite rice variety MR263 through marker-assisted backcrossing. J Sci Food Agric. 2016. doi:10.1002/jsfa.7222

50. Suh JP, Jeung JU, Noh TH, Cho YC, Park SH, Park HS, et al. Development of breeding lines with three pyramided resistance genes that confer broad-spectrum bacterial blight resistance and their molecular analysis in rice. Rice. 2013;6: 1–11. doi:10.1186/1939-8433-6-5

51. Suh JP, Jeung JU, Noh TH, Cho YC, Park SH, Park HS, et al. Development of breeding lines with three pyramided resistance genes that confer broad-spectrum bacterial blight resistance and their molecular analysis in rice. Rice. 2013. doi:10.1186/1939-8433-6-5

52. Arunakumari K, Durgarani C V., Satturu V, Sarikonda KR, Chittoor PDR, Vutukuri B, et al. Marker-Assisted Pyramiding of Genes Conferring Resistance Against Bacterial Blight and Blast Diseases into Indian Rice Variety MTU1010. Rice Sci. 2016;23: 306–316. doi:10.1016/J.RSCI.2016.04.005

53. Pradhan KC, Pandit E, Mohanty SP, Moharana A, Sanghamitra P, Meher J, et al. Development of Broad Spectrum and Durable Bacterial Blight Resistant Variety through Pyramiding of Four Resistance Genes in Rice. Agron 2022, Vol 12, Page 1903. 2022;12: 1903. doi:10.3390/AGRONOMY12081903

54. Hasan MM, Rafii MY, Ismail MR, Mahmood M, Rahim HA, Alam MA, et al. Marker-assisted backcrossing: A useful method for rice improvement. Biotechnol Biotechnol Equip. 2015. doi:10.1080/13102818.2014.995920

55. Chukwu SC, Rafii MY, Ramlee SI, Ismail SI, Oladosu Y, Muhammad I, et al. Recovery of Recurrent Parent Genome in a Marker-Assisted Backcrossing Against Rice Blast and Blight Infections Using Functional Markers and SSRs. Plants 2020, Vol 9, Page 1411. 2020;9: 1411. doi:10.3390/PLANTS9111411

56. Lü L, Liu S, Jiang S, Wu S. Tannin inhibits HIV-1 entry by targeting gp41. Acta Pharmacol Sin. 2004;25: 213–8. Available: http://www.ncbi.nlm.nih.gov/pubmed/14769212

57. Ramalingam J, Savitha P, Alagarasan G, Saraswathi R, Chandrababu R. Functional marker assisted improvement of stable cytoplasmic male sterile lines of rice for bacterial blight resistance. Front Plant Sci. 2017. doi:10.3389/fpls.2017.01131

58. Perumalsamy S, Bharani M, Sudha M, Nagarajan P, Arul L, Saraswathi R, et al. Functional marker-assisted selection for bacterial leaf blight resistance genes in rice (Oryza sativa L.). Plant Breed. 2010;129: 400–406. doi:10.1111/J.1439-0523.2009.01705.X

59. Dokku P, Das KM, Rao GJN. Pyramiding of four resistance genes of bacterial blight in Tapaswini, an elite rice cultivar, through marker-assisted selection. Euphytica. 2013;192: 87–96. doi:10.1007/S10681-013-0878-2/METRICS

60. Pradhan SK, Nayak DK, Pandit E, Behera L, Anandan A, Mukherjee AK, et al. Incorporation of Bacterial Blight Resistance Genes Into Lowland Rice Cultivar Through Marker-Assisted Backcross Breeding. Phytopathology. 2016;106: 710–718. doi:10.1094/PHYTO-09-15-0226-R

61. Pradhan SK, Pandit E, Pawar S, Baksh SY, Mukherjee AK, Mohanty SP. Development of flash-flood tolerant and durable bacterial blight resistant versions of mega rice variety ‘Swarna’ through marker-assisted backcross breeding. Sci Reports 2019 91. 2019;9: 1–15. doi:10.1038/s41598-019-49176-z

62. Chukwu SC, Rafii MY, Ramlee SI, Ismail SI, Hasan MM, Oladosu YA, et al. Bacterial leaf blight resistance in rice: a review of conventional breeding to molecular approach. Molecular Biology Reports. 2019. doi:10.1007/s11033-019-04584-2

63. Zhan XD, Zhou HP, Chai RY, Zhuang JY, Cheng SH, Cao LY. Breeding of R8012, a Rice Restorer Line Resistant to Blast and Bacterial Blight Through Marker-Assisted Selection. Rice Sci. 2012;19: 29–35. doi:10.1016/S1672-6308(12)60017-1

64. Huang JZ, E ZG, Zhang HL, Shu QY. Workable male sterility systems for hybrid rice: Genetics, biochemistry, molecular biology, and utilization. Rice. 2014;7: 1–14. doi:10.1186/S12284-014-0013-6

65. Balachiranjeevi CH, Bhaskar NS, Abhilash V, Akanksha S, Viraktamath BC, Madhav MS, et al. Marker-assisted introgression of bacterial blight and blast resistance into DRR17B, an elite, fine-grain type maintainer line of rice. Mol Breed. 2015;35: 1–12. doi:10.1007/S11032-015-0348-8/METRICS

Fig. 1

Click here to access/download;Figure;Figure 1.tif

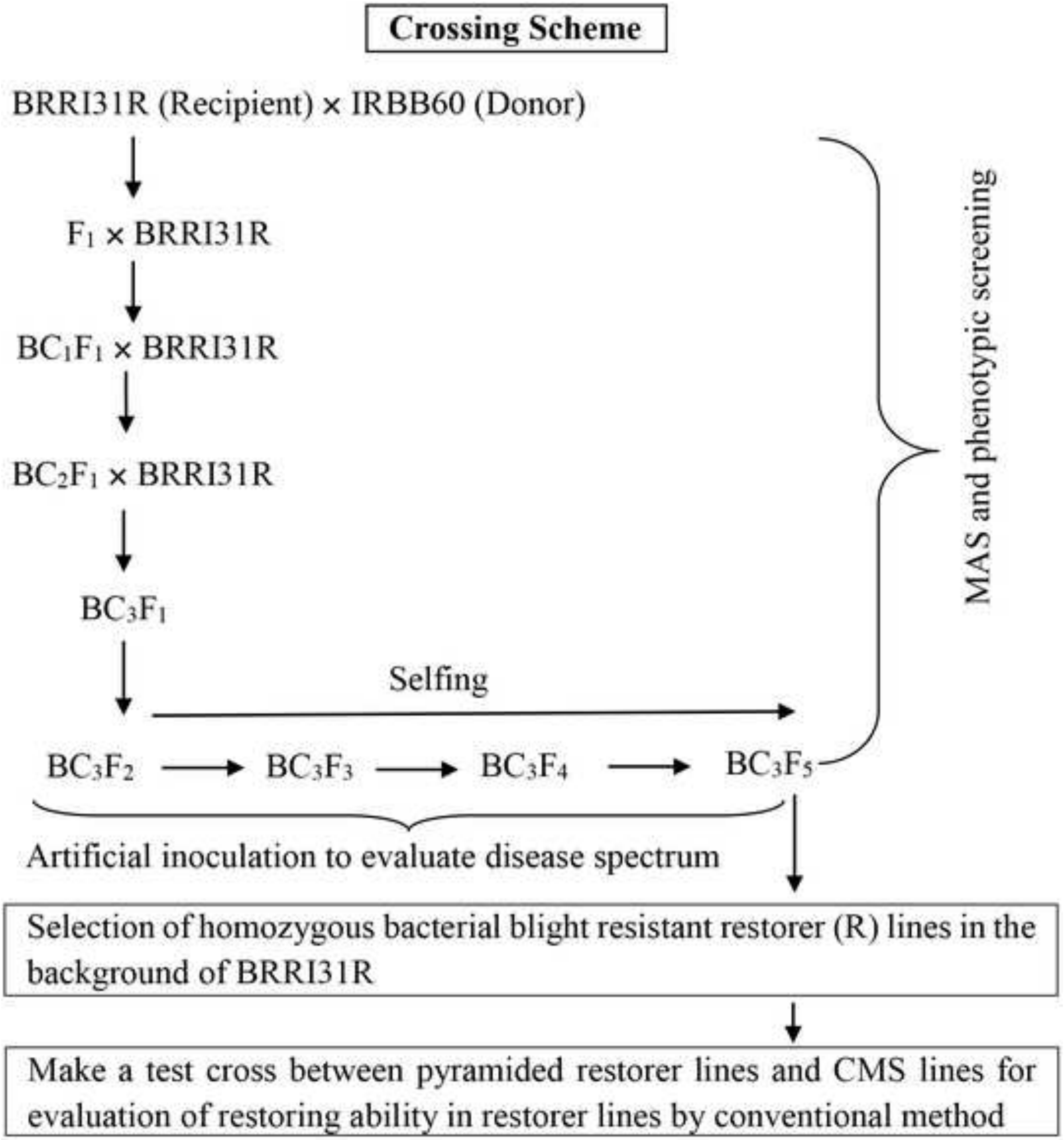

Fig. 2

Click here to access/download;Figure;Figure 2.tif

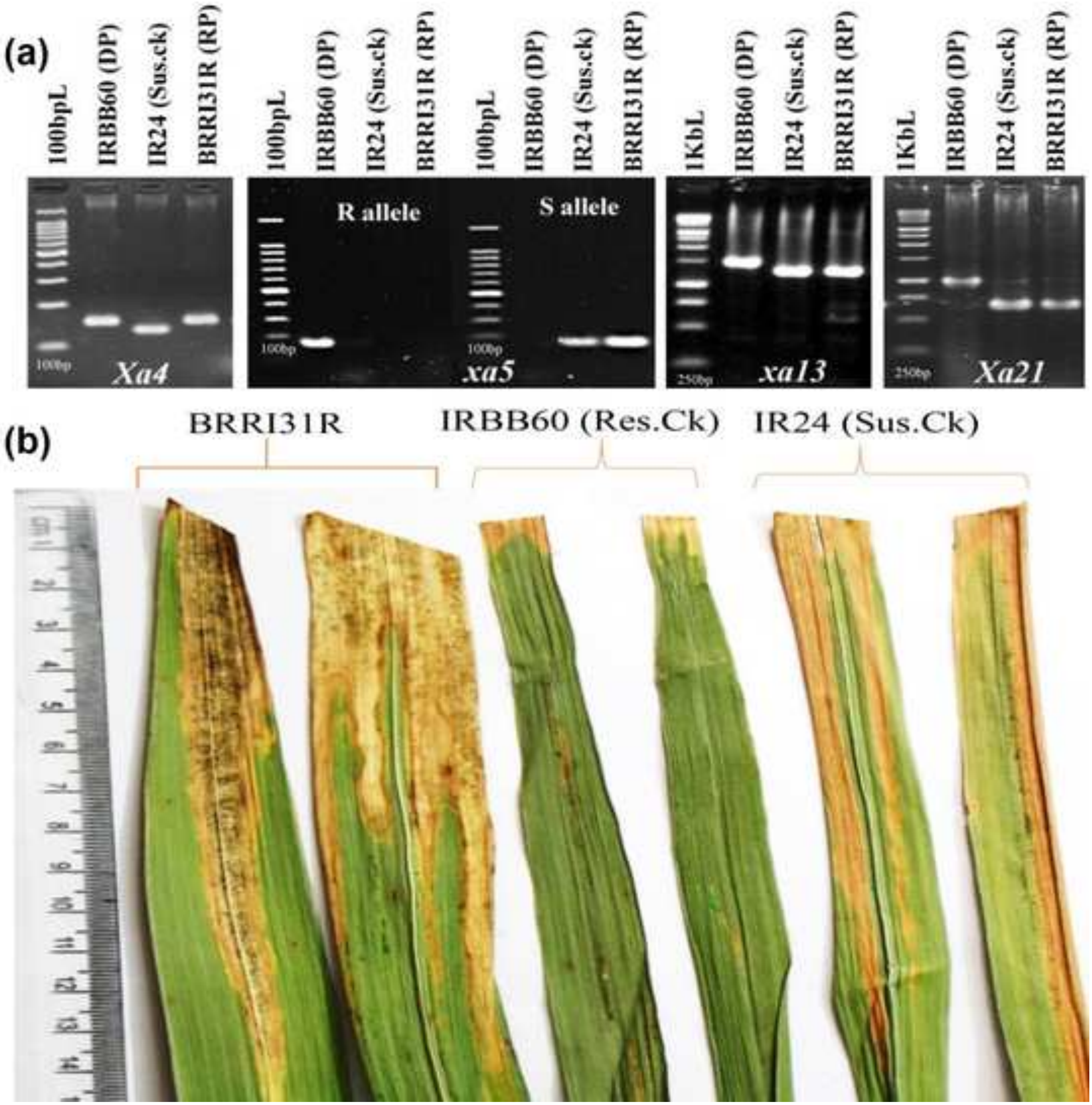

Fig. 3

Click here to access/download;Figure;Figure 3.tif

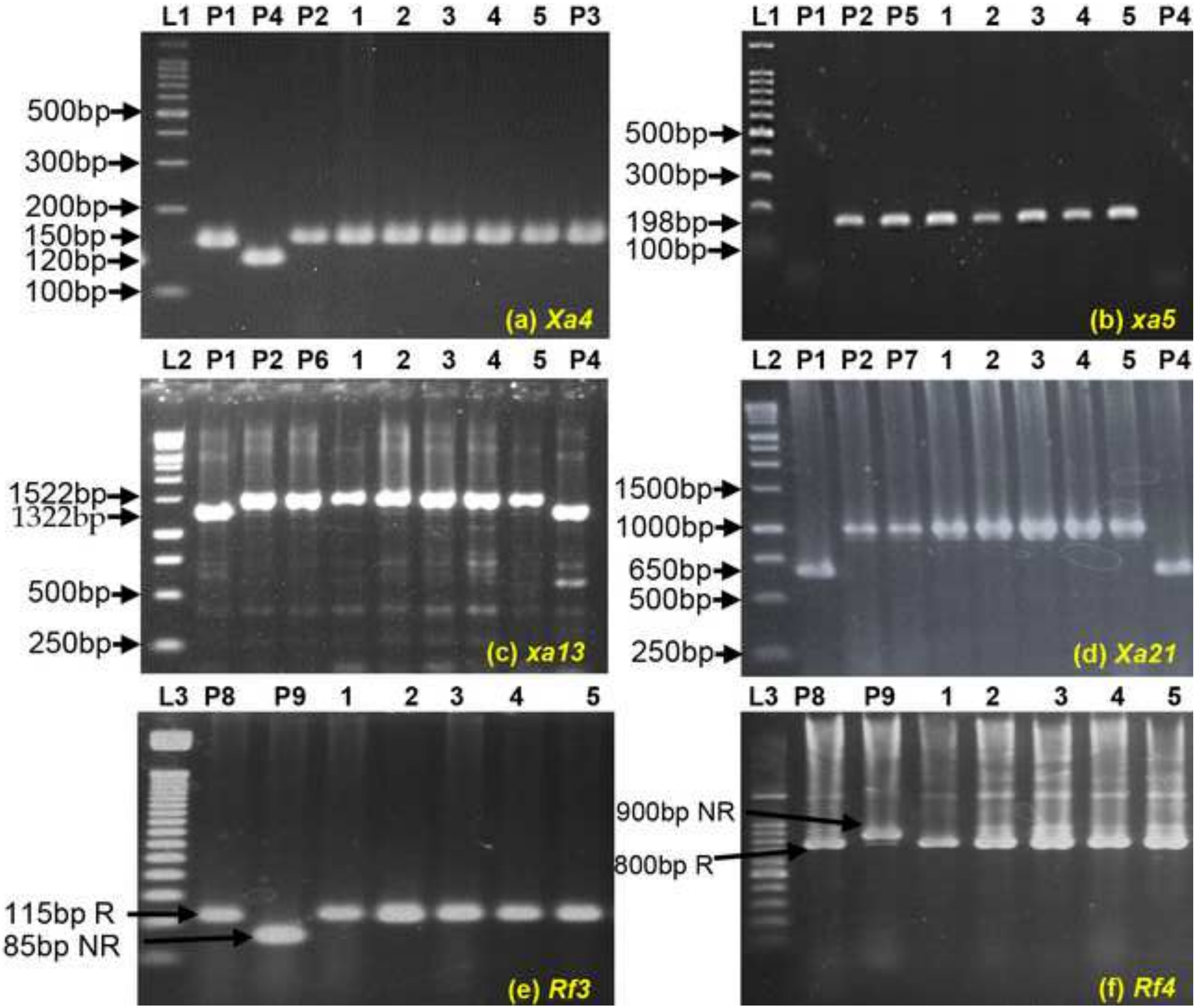

Fig. 4

Click here to access/download;Figure;Figure 4.tif

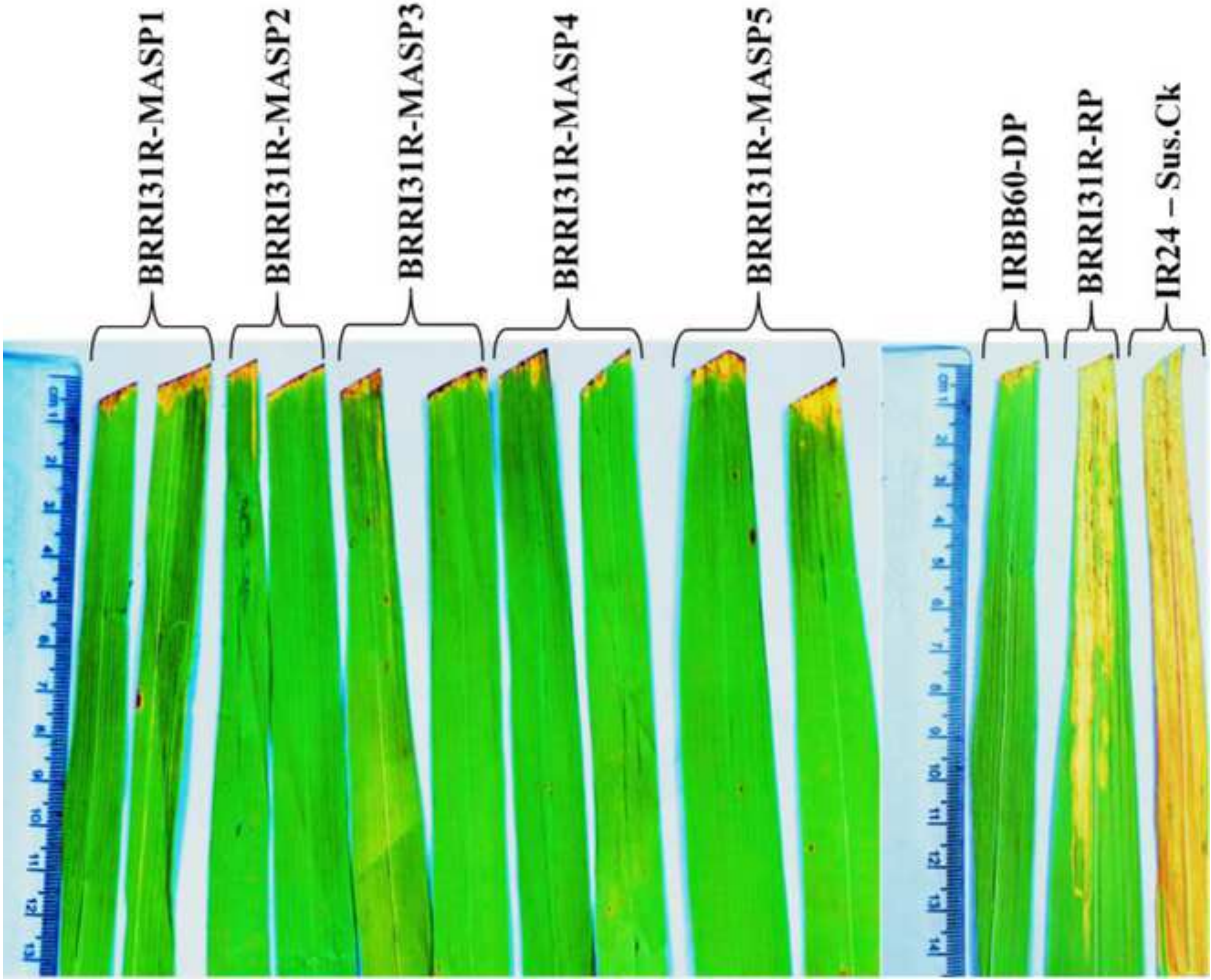

Fig. 5

Click here to access/download;Figure;Figure 5.tif

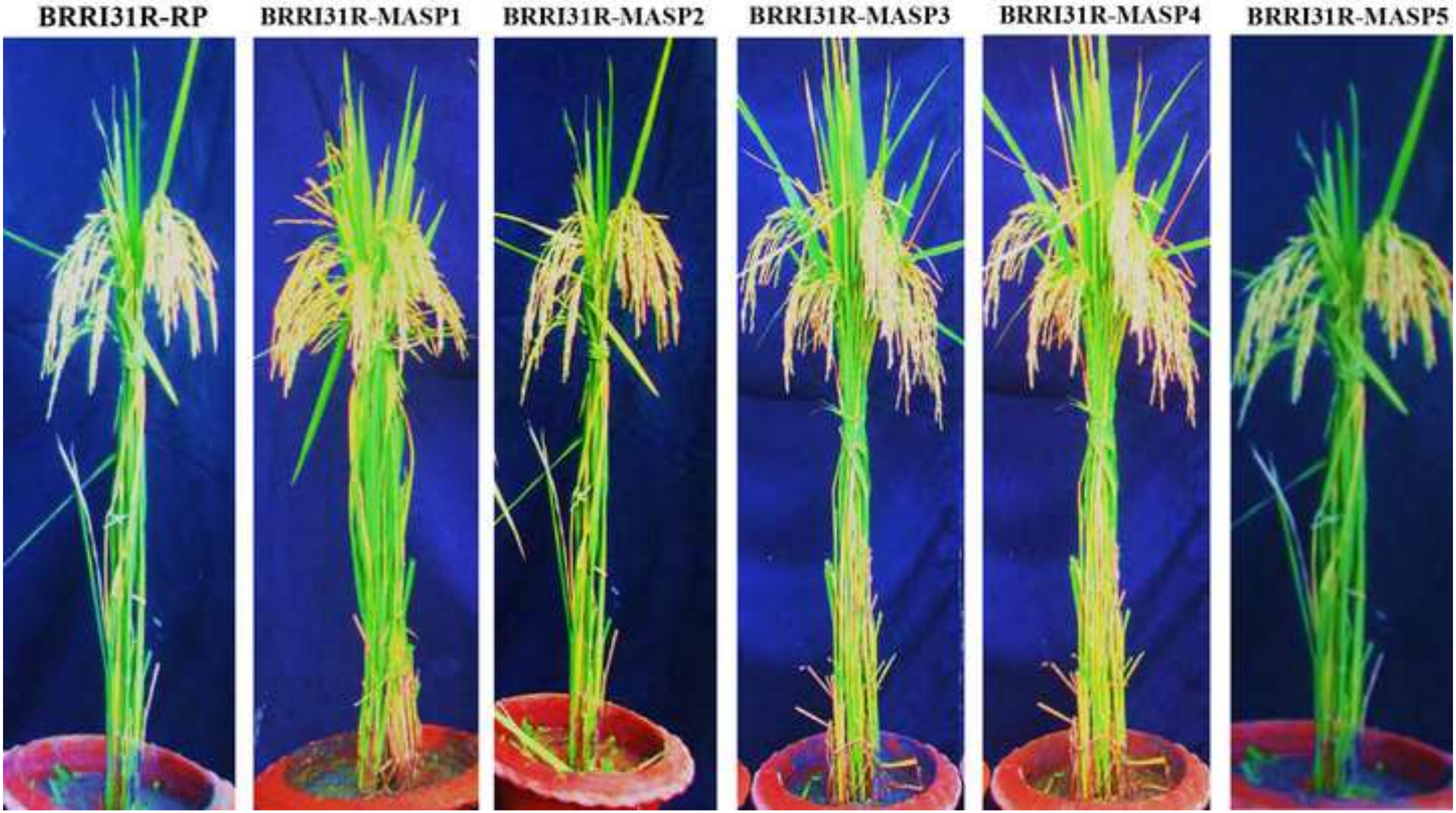

Click here to access/download
Supporting Information
Supplementary table.docx
