# Supplementary material for: Pyramiding of bacterial blight resistance genes into promising restorer BRRI31R line through marker-assisted backcross breeding and evaluation of agro-morphological and physiochemical characteristics of developed resistant restorer lines
Source: PLoS One. 2024 Jun 12;19(6):e0301342. doi: 10.1371/journal.pone.0301342 (PMC11168670; doi:10.1371/journal.pone.0301342)
Supplement: Attachment — Submitted filename: Response to reviewers comments.docx [file pone.0301342.s007.docx]

**Response to Editor and Reviewers comments**

**Journal requirements:**

**When submitting your revision, we need you to address these additional requirements.**

**Comment 1.** Please ensure that your manuscript meets PLOS ONE's style requirements, including those for file naming. The PLOS ONE style templates can be found at

https://journals.plos.org/plosone/s/file?id=wjVg/PLOSOne_formatting_sample_main_body.pdf and

https://journals.plos.org/plosone/s/file?id=ba62/PLOSOne_formatting_sample_title_authors_affiliations.pdf

Response: We checked and tried to arrange the manuscript according to PLOS one journal format.

**Comment 2**. We suggest you thoroughly copyedit your manuscript for language usage, spelling, and grammar. If you do not know anyone who can help you do this, you may wish to consider employing a professional scientific editing service.

Whilst you may use any professional scientific editing service of your choice, PLOS has partnered with both American Journal Experts (AJE) and Editage to provide discounted services to PLOS authors. Both organizations have experience helping authors meet PLOS guidelines and can provide language editing, translation, manuscript formatting, and figure formatting to ensure your manuscript meets our submission guidelines. To take advantage of our partnership with AJE, visit the AJE website (http://learn.aje.com/plos/) for a 15% discount off AJE services. To take advantage of our partnership with Editage, visit the Editage website (www.editage.com) and enter referral code PLOSEDIT for a 15% discount off Editage services. If the PLOS editorial team finds any language issues in text that either AJE or Editage has edited, the service provider will re-edit the text for free.

Response: We tried to revise the language of our manuscript. The manuscript has been edited by Dr. Mohammad Abdul Latif, Director, Admin and Common Service and Former Head and Chief Scientific Officer of the Plant Pathology Division of Bangladesh Rice Research Institute (BRRI), Gazipur, Bangladesh.

**Comment 3**. Thank you for stating the following financial disclosure:

“Authors received funds from Strengthening Physical Infrastructure and Research Activities (SPIRA) Project, Bangladesh Rice Research Institute, through the Ministry of Agriculture, Bangladesh to conduct this experiment.”

Please state what role the funders took in the study. If the funders had no role, please state: "The funders had no role in study design, data collection and analysis, decision to publish, or preparation of the manuscript."

If this statement is not correct you must amend it as needed.

Please include this amended Role of Funder statement in your cover letter; we will change the online submission form on your behalf.

Response: We already included the statement "The funders had no role in study design, data collection and analysis, decision to publish, or preparation of the manuscript” in the funding section.

**Comment 4**. Thank you for stating the following in the Acknowledgments Section of your manuscript:

“The authors would like to express their gratitude to the Project Director for the financial assistance provided by the Strengthening Physical Infrastructure and Research Activities (SPIRA) Project, Bangladesh Rice Research Institute, through the Ministry of Agriculture, Bangladesh. The authors are highly grateful to Plant Pathology Division and Hybrid Rice Division for providing all necessary support. The authors are extremely thankful to the Plant Pathology Division and the Hybrid Rice Division for providing all required assistance.”

We note that you have provided funding information that is currently declared in your Funding Statement. However, funding information should not appear in the Acknowledgments section or other areas of your manuscript. We will only publish funding information present in the Funding Statement section of the online submission form.

Please remove any funding-related text from the manuscript and let us know how you would like to update your Funding Statement. Currently, your Funding Statement reads as follows:

“Authors received funds from Strengthening Physical Infrastructure and Research Activities (SPIRA) Project, Bangladesh Rice Research Institute, through the Ministry of Agriculture, Bangladesh to conduct this experiment.”

Please include your amended statements within your cover letter; we will change the online submission form on your behalf.

Response: We removed the funding information from the Acknowledgement section and updated the funding statement in the Funding section.

**Comment 5**. When completing the data availability statement of the submission form, you indicated that you will make your data available on acceptance. We strongly recommend all authors decide on a data sharing plan before acceptance, as the process can be lengthy and hold up publication timelines. Please note that, though access restrictions are acceptable now, your entire data will need to be made freely accessible if your manuscript is accepted for publication. This policy applies to all data except where public deposition would breach compliance with the protocol approved by your research ethics board. If you are unable to adhere to our open data policy, please kindly revise your statement to explain your reasoning and we will seek the editor's input on an exemption. Please be assured that, once you have provided your new statement, the assessment of your exemption will not hold up the peer review process.

Response: We attached our data as a supporting file. Please check.

**Comment 6**. PLOS ONE now requires that authors provide the original uncropped and unadjusted images underlying all blot or gel results reported in a submission’s figures or Supporting Information files. This policy and the journal’s other requirements for blot/gel reporting and figure preparation are described in detail at https://journals.plos.org/plosone/s/figures#loc-blot-and-gel-reporting-requirements and https://journals.plos.org/plosone/s/figures#loc-preparing-figures-from-image-files. When you submit your revised manuscript, please ensure that your figures adhere fully to these guidelines and provide the original underlying images for all blot or gel data reported in your submission. See the following link for instructions on providing the original image data: https://journals.plos.org/plosone/s/figures#loc-original-images-for-blots-and-gels.

In your cover letter, please note whether your blot/gel image data are in Supporting Information or posted at a public data repository, provide the repository URL if relevant, and provide specific details as to which raw blot/gel images, if any, are not available. Email us at plosone@plos.org if you have any questions.

Response: We provided the raw images as supporting information.

**Comment 7**. Please include captions for your Supporting Information files at the end of your manuscript, and update any in-text citations to match accordingly. Please see our Supporting Information guidelines for more information: http://journals.plos.org/plosone/s/supporting-information.

Response: Included the captions for Supporting Information files and cited in the text accordingly.

**Comment 8**. Please review your reference list to ensure that it is complete and correct. If you have cited papers that have been retracted, please include the rationale for doing so in the manuscript text, or remove these references and replace them with relevant current references. Any changes to the reference list should be mentioned in the rebuttal letter that accompanies your revised manuscript. If you need to cite a retracted article, indicate the article’s retracted status in the References list and also include a citation and full reference for the retraction notice.

Response: Reviewed the references list and tried to correct it according to PLOSOne Journal style.

**Additional Editor Comments:**

The manuscript was well written and interesting. However, there are some minor clarification requirement from the authors.

**Comment 9**. What is the concentration of bacterial cell for inoculation on parent and selected pyramided restorer lines?

Response: Bacterial cell concentration was 3.3 × 10^8^ CFU/mL and the OD_600_ value was 1, which is equivalent to this concentration.

**Comment 10**. In Table 2, RP, DP, RC and Sc were mentioned. What are they?

Response: Updated in the Table 2. RP means Recipient parent; DP, Donor parent; RC, Resistant check; Sc, Susceptible check.

**Comment 11**. The suggestions of the reviewer (2) may be incorporated in the revised manuscript.

[Note: HTML markup is below. Please do not edit.]

**Reviewers' comments:**

**Reviewer's Responses to Questions**

Comments to the Author

1. Is the manuscript technically sound, and do the data support the conclusions?

The manuscript must describe a technically sound piece of scientific research with data that supports the conclusions. Experiments must have been conducted rigorously, with appropriate controls, replication, and sample sizes. The conclusions must be drawn appropriately based on the data presented.

Reviewer #1: No

Reviewer #2: Yes

2. Has the statistical analysis been performed appropriately and rigorously?

Reviewer #1: Yes

Reviewer #2: Yes

3. Have the authors made all data underlying the findings in their manuscript fully available?

The PLOS Data policy requires authors to make all data underlying the findings described in their manuscript fully available without restriction, with rare exception (please refer to the Data Availability Statement in the manuscript PDF file). The data should be provided as part of the manuscript or its supporting information, or deposited to a public repository. For example, in addition to summary statistics, the data points behind means, medians and variance measures should be available. If there are restrictions on publicly sharing data—e.g. participant privacy or use of data from a third party—those must be specified.

Reviewer #1: Yes

Reviewer #2: Yes

4. Is the manuscript presented in an intelligible fashion and written in standard English?

PLOS ONE does not copyedit accepted manuscripts, so the language in submitted articles must be clear, correct, and unambiguous. Any typographical or grammatical errors should be corrected at revision, so please note any specific errors here.

Reviewer #1: Yes

Reviewer #2: Yes

**Review Comments to the Author**

Please use the space provided to explain your answers to the questions above. You may also include additional comments for the author, including concerns about dual publication, research ethics, or publication ethics. (Please upload your review as an attachment if it exceeds 20,000 characters)

**Reviewer #1:**

**Comment 12.** I appreciate the author who has taken the effort since It has been a burning issue in rice cultivation at this time since the Rice (Oryza sativa L.) is the world's most influential and economically significant crop.

Response: Thank you for your nice complements.

**Reviewer #2:**

The manuscript by Akter and colleagues focuses on the pyramiding of four bacterial blight resistance genes in the background of BRRI31R through marker-assisted backcross breeding. The manuscript is well written, and the figures are well presented. While the topic will have broad readers, are few minor revisions are required. As such, I would recommend important changes to be taken into account in the manuscript before acceptance for publication.

Response: We are very much grateful for your valuable comments. We tried to answer your queries in the following section.

**Comments/queries that need to be addressed:**

**Comment 13.** The author claims that restorer line BRRI31R carries the Xa4 gene in the background based on the amplification of resistant allele corresponding to donor parent IRBB60 and resistant check IRBB4. In this regard, please consider the following points: (a) Please provide the parentage of BRRI31R if the Xa4 has been transferred from the IRBB4. (b) Kindly mention if the marker is functional and also provide the information on the response of IRBB4.

Response: BR7013-62-1-1R X BRRI20R is the true parentage of BRRI31R. From this cross combination, the fixed line, HRB167-19-7-7-1R, was discovered and it registered is BRRI31R. In addition, we don’t know from where Xa4 is inbuilt in the inbred lines of Bangladesh but we tested that Xa4 is not working in Bangladesh by using the monogenic lines IRRBB4 (check the Table 1 of below reference 1). For your kind concern, we used the popular functional marker to identify the Xa4 gene. Please check the following paper (2 and 3) for Xa4 marker.

1.Rashid MM, Nihad SAI, Khan MAI, et al (2021) Pathotype profiling, distribution and virulence analysis of Xanthomonas oryzae pv. oryzae causing bacterial blight disease of rice in Bangladesh. J Phytopathol 169:438–446. <https://onlinelibrary.wiley.com/doi/abs/10.1111/jph.13000>

2.Dossa GS, Oliva R, Maiss E, Vera Cruz C, Wydra K. High Temperature Enhances the Resistance of Cultivated African Rice, Oryza glaberrima, to Bacterial Blight. Plant Dis. 2016 Feb;100(2):380-387. doi: [10.1094/PDIS-05-15-0536-RE](https://pubmed.ncbi.nlm.nih.gov/30694136/). Epub 2015 Dec 19. PMID: 30694136.

3.Suh JP, Jeung JU, Noh TH, Cho YC, Park SH, Park HS, Shin MS, Kim CK, Jena KK. Development of breeding lines with three pyramided resistance genes that confer broad-spectrum bacterial blight resistance and their molecular analysis in rice. Rice (N Y). 2013 Feb 8;6(1):5. doi: [10.1186/1939-8433-6-5](https://pubmed.ncbi.nlm.nih.gov/24280417/). PMID: 24280417; PMCID: PMC4883717.

**Comment 14.** The author tested 200 BC3F2 with linked markers and identified homozygous plants with single, double, and triple gene combinations. Please mention if the author has checked the effectiveness and spectrum of various resistance gene combinations against five Xoo races of Bangladesh to ensure its effectiveness/durability without having negative impacts on other agronomic traits.

Response: Yes, we checked the studied resistance genes against the five Xoo races. Moreover, in separate studies, we also tested the efficacy of the studied genes against the studied race and other virulent races of Bangladesh. Please see the following references. However, we have verified the efficacy and range of different resistance gene combinations and it does not adversely affect other agronomic characteristics.

1.Rashid MM, Nihad SAI, Khan MAI, et al (2021) Pathotype profiling, distribution and virulence analysis of Xanthomonas oryzae pv. oryzae causing bacterial blight disease of rice in Bangladesh. J Phytopathol 169:438–446. <https://onlinelibrary.wiley.com/doi/abs/10.1111/jph.13000>

2. Anik TR, Nihad SAI, Hasan MA-I, et al (2022) Exploring of bacterial blight resistance in landraces and mining of resistant gene(s) using molecular markers and pathogenicity approach. Physiol Mol Biol Plants 2022 282 28:455–469. <https://link.springer.com/article/10.1007/s12298-022-01139-x>

**Comment 15.** It is important to mention the initial population size for BC3F2. The 200 is a very small population to select the combination of four genes. The chances of escape of the gene are high in this population. Additionally, the selection of a superior, high-yielding, early maturing line in such a small is difficult.

Response: Yes, we are aware that the 200 BC3F2 population is really small, but fortunately, we was able to obtain homozygous lines that were highly similar to the recurrent parent and carried four BB resistance genes (Xa4, Xa5, Xa13 and Xa21).

**Comment 16.** The pollen fertility of IR79156A x BRRI31R-MASP2 and IR79156A x BRRI31R-MASP5 is 91.95 and 93.33, respectively. But why they are not considered for further analysis?

Response: This study comes from a doctoral dissertation of Anowara Akter (first author). She is also a researcher of Hybrid Rice Division of Bangladesh Rice Research Institute. She is continuing her research. There is no Hybrid line resistance against the BB pathogen of Bangladesh. As a result, she introduced BB resistance genes into the Maintainer Line and later she will transfer these genes into the corresponding CMS line to develop BB resistant hybrid variety. So her research is still ongoing.

**Comment 17.** Why two pyramiding lines MASP3 and MASP4 are selectively biased for having improved agronomic traits over the other three pyramided lines as evidenced by Table 7 and Table 8? Similarly, for instance, Table 4 shows the mean lesion length of these two lines very similar to the donor over the other three pyramided lines and they carry the pita gene as well.

Response: Thank you for your valuable comments. MASP3 and MASP4 are highly resistant against the BB races. Mean lesion length of MASP3 and MASP4 were 0.3 and 0.2 cm, respectively (Table 4). So we selected these two lines. Additionally, As this work derived from a PhD work, so it was not possible to go with all the lines for further analysis.

**Comment 18.** Line 254- A gel photograph…. It seems that the sentence is incomplete. Similarly,

Response: We are sorry for the mistake We removed the incomplete sentence.

**Comment** **19.** The reference Suh et al. 2013 is repeated twice (50 and 51).

Response: Removed the duplicate one.

**Comment 20.** Check the reference 56 if it is relevant.

Response: Removed the reference.

**Comment 21.** PLOS authors have the option to publish the peer review history of their article (what does this mean?). If published, this will include your full peer review and any attached files.

Response: We have no problem to publish the peer review history.

If you choose “no”, your identity will remain anonymous but your review may still be made public.

Do you want your identity to be public for this peer review? For information about this choice, including consent withdrawal, please see our Privacy Policy.

Reviewer #1: Yes: Dr. Ayyasami Ramanathan, Professor-Plant Pathology, Tamilnadu Agricultural University, Thanjavur, Tamilnadu, India

Reviewer #2: Yes: Dr. Kishor Kumar

**Comment 22.** While revising your submission, please upload your figure files to the Preflight Analysis and Conversion Engine (PACE) digital diagnostic tool, https://pacev2.apexcovantage.com/. PACE helps ensure that figures meet PLOS requirements. To use PACE, you must first register as a user. Registration is free. Then, login and navigate to the UPLOAD tab, where you will find detailed instructions on how to use the tool. If you encounter any issues or have any questions when using PACE, please email PLOS at figures@plos.org. Please note that Supporting Information files do not need this step.

Response: We checked the quality of our images through PACE.
